# Applying Isothermal Titration Calorimetry and Saturation Transfer Difference-NMR to Study the Mode of Interaction of Flavan-3-ols with α-Amylase to Understand Their Impact on Starch Hydrolysis

**DOI:** 10.1021/acs.jafc.4c13178

**Published:** 2025-04-04

**Authors:** Birgit Claasen, Mengyao Xiong, Pia S. Mayer, Greta Sogl, Maria Buchweitz

**Affiliations:** 1 Analytical Department, Institute of Organic Chemistry, University of Stuttgart, Pfaffenwaldring 55, Stuttgart 70569, Germany; 2 Department of Food Chemistry, Institute of Biochemistry and Technical Biochemistry, University of Stuttgart, Allmandring 5b, Stuttgart 70569, Germany; 3 Institute of Food Chemistry, University of Hamburg, Martin-Luther-King-Platz 6, Hamburg 20146, Germany

**Keywords:** procyanidins, proton signal assignment, rotamers, circular dichroism spectroscopy, enzyme inhibition, digestive enzymes, enzyme activity assay

## Abstract

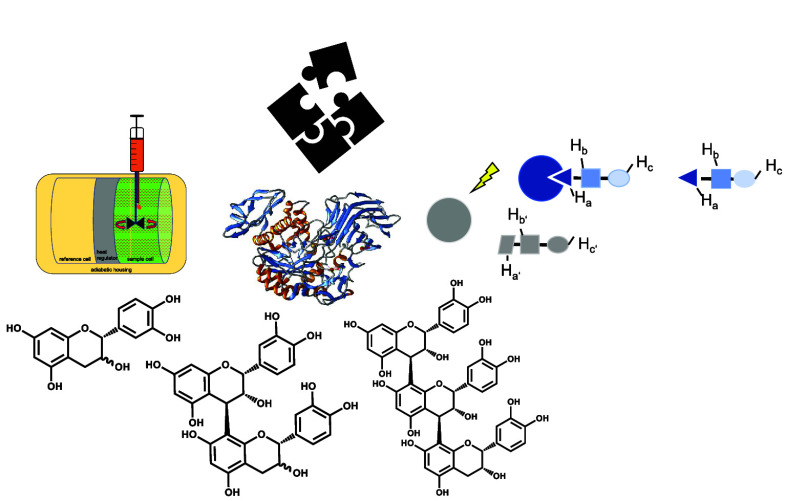

For flavan-3-ols, significant effects to prevent the development of diabetes mellitus are postulated. *Inter alia*, this is attributed to inhibitory effects on the intestinal α-amylase, in particular for high-molecular-weight procyanidins. In order to gain a deeper insight into the mode of interaction and the resulting α-amylase inhibition, the interaction between the monomers (+)-catechin (CAT) and (−)-epicatechin (EC), the dimers procyanidin (PC) B1 and PC B2, and the trimer PC C1 and their inhibition of porcine pancreatic α-amylase were investigated. Weak interactions were determined by isothermal titration calorimetry (ITC), with no clear difference between monomers and dimers and even no observable interaction with PC C1. Data from saturation transfer difference (STD)-NMR experiments supported these results with respect to reversible interactions. The detailed NMR signal assignments revealed that the formation of rotamers is solvent-dependent, which might explain the differences in the interaction strength between both diastereomers. The results for interaction were in contrast to the accumulating inhibitory strength with an increasing degree of polymerization when monitoring hydrolysis of the natural substrate starch in a novel continuous approach by ITC. By combining the data from the interaction and inhibition studies, we propose that protein aggregation occurs in the presence of flavan-3-ol oligomers, which are responsible for the inhibitory effects. This rather irreversible interaction is not susceptible to detection by ITC and STD-NMR and was also not observable by CD spectroscopy.

## 1 Introduction

The development of type 2 diabetes and cardiovascular diseases is frequently associated with postprandial hyperglycemia, which is defined as elevated blood glucose levels, particularly following the consumption of food. Consequently, the inhibition of intestinal α-amylase and α-glucosidase reduces the conversion of starch to malto-oligosaccharides and glucose, thereby preventing spikes in blood glucose levels.^1,2^ For example, acarbose, a well-known artificial α-amylase and α-glucosidase inhibitor, is routinely applied to patients suffering from type 2 diabetes, to treat high blood glucose concentration.^3^ In this context, polyphenols have been explored as a natural alternative to prevent the development of postprandial hyperglycemia.^1,4−7^ In addition, it has also been debated that interaction of polyphenols with starch might result in its limited digestibility and therefore reduced glucose formation.^8^

**Figure 1:**
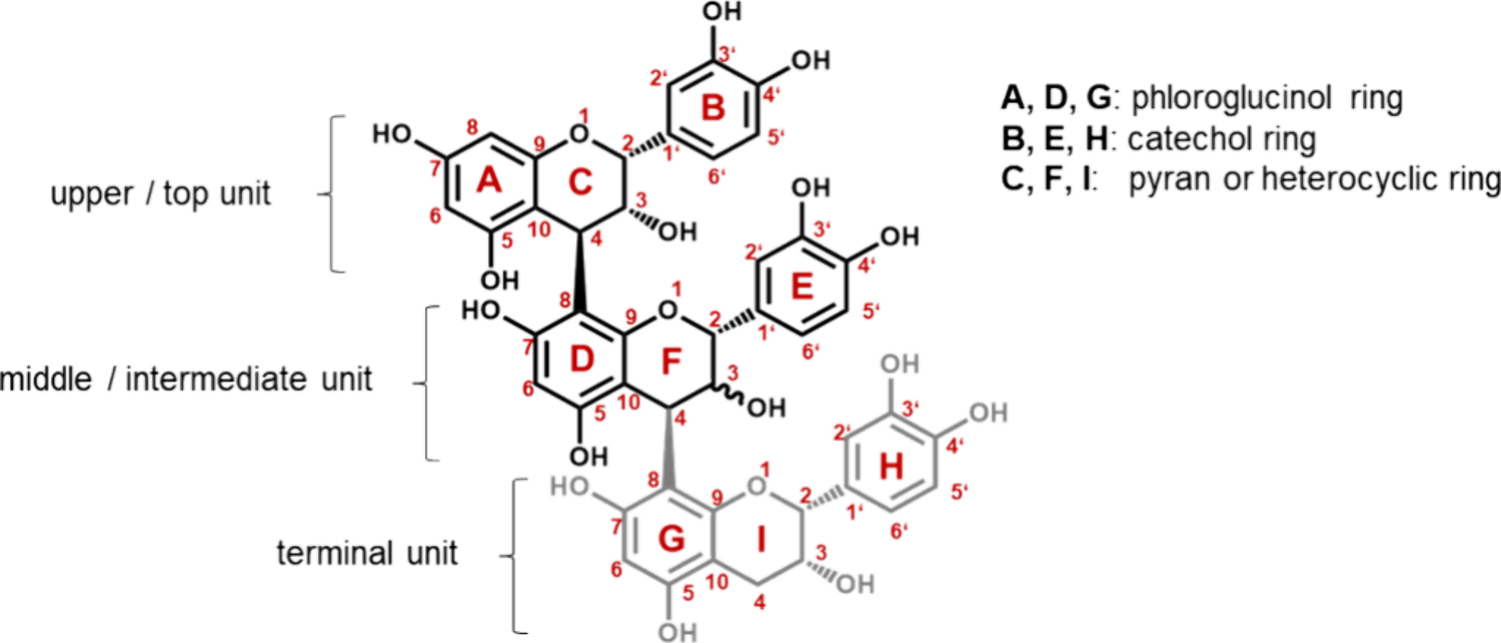
Structures for procyanidins and typical nomenclatures.^18−20^ PC B1 (EC-CAT) and PC B2 (EC-EC) are dimers and diastereomers. In PC B1, the hydrogen atom on carbon F3 points to the front side of the plane (stereocenter S), while the hydrogen atom on carbon F3 in PC B2 is facing the backside of the plane (stereocenter R). PC C1 (EC-EC-EC) is a trimer with the hydrogen atom on carbon F3 on the backside of the plane.

One subgroup of the polyphenols is flavan-3-ols covering the monomers (+)-catechin (CAT) and (−)-epicatechin (EC) as well as their di- and oligomeric structures (procyanidins: PC B1, PC B2, and PC C1; Figure 1), which have high potential to interact with proteins, polysaccharides, and surfaces of bacteria and viruses.^9−13^ Depending on the linkage between the monomer units, flavan-3-ol dimers are categorized into the A-type and B-type. B-type procyanidins are most abundant and characterized by a single interflavan bond between carbon C4 (upper unit) and either carbon D8 or D6 (lower unit).^14,15^ A-type procyanidins exhibit an additional ether linkage between carbon C2 and the hydroxyl group at carbon D7. Procyanidins contain 2–7 monomeric units and are categorized by their degree of polymerization (DP); however, dimers and trimers are the most studied structures due to limitations in extraction of structures with a higher DP and challenges in structure elucidation.^12,16,17^

The interaction of flavan-3-ols with proteins has been widely studied, demonstrating a high impact of the individual structure according to the molecular weight (DP), steric demand, flexibility, and the number of hydroxyl and further functional groups.^12,21^ For example, the degree of galloylation is a strong indicator for higher protein affinities and a linkage from carbon C4 to D8 results in higher binding affinities than an interflavan bond from carbon C4 to D6.^16,18^ Flavanol solubility and concentration are further important impact factors.^22^

The interaction of polyphenols with proteins might result in the formation of covalent bonds due to oxidation to quinones. These highly reactive intermediates can undergo nucleophilic attacks by protein side chains (e.g., lysine, serine, cysteine, and tyrosine).^11,12,22,23^ However, noncovalent interactions play a decisive role, with protein mobility and flexibility representing crucial parameters for this mechanism. The hydrophobic nature of phenolics enables them to interact with hydrophobic amino acid side chains and aromatic residues, such as phenylalanine and tyrosine. Furthermore, their hydroxyl groups might form hydrogen bonds with the polar functional groups of the proteins (e.g., amide, amino, guanidine, and carboxyl groups).^10,12,24,25^

For α-amylase (57 kDa), inhibition of the enzyme activity via an interaction with various phenolic compounds has been reported.^26^ However, far too little attention has been paid to the flavan-3-ol subgroup, despite its great structural and steric diversity. A few studies have investigated the interaction strength and kinetics of procyanidins to inhibit digestive enzymes and found a strong influence of the degree of polymerization (DP). For example, Gu et al. performed a comprehensive kinetic study on the inhibition of digestive enzymes. While the effect of cocoa procyanidins on lipases and phospholipases was marked, their effect on α-amylase activity was less pronounced.^27^ In general, the interaction has been studied mainly by fluorescence quenching, which is not sufficient to distinguish between reversible and irreversible interactions.^4,1,28,5^ Furthermore, α-amylase activity assays have been performed using artificial substrates, where hydrolysis is detected by UV/Vis and fluorescence spectroscopy at discrete time intervals or end points. They therefore do not allow continuous monitoring of the complete starch hydrolysis over time.

The aim of the present study was to investigate the effect of DP of flavan-3-ol on the interaction mechanism with intestinal (porcine) α-amylase using direct and label-free approaches such as isothermal titration calorimetry (ITC) and saturation transfer difference-NMR (STD-NMR), and to correlate the data with the resulting inhibition of starch hydrolysis.

ITC provides a comprehensive thermodynamic profile (i.e., equilibrium dissociation constants *K*_D_, enthalpy changes Δ*H*, entropy changes Δ*S*, and Gibbs free energy changes Δ*G*) of an interaction, as well as the ligand:protein ratio (i.e., stoichiometry *n*) when a specific interaction occurs, based on the heat release during the multiple injection of a ligand to a protein.^29^ In the STD-NMR experiment, the protein is selectively saturated by a pulse, and in the event of ligand binding, magnetization is transferred from the protein to the binding protons of the ligand.^30,31^ An off-resonance spectrum, in the absence of a saturation pulse, and an on-resonance spectrum are acquired. The STD spectrum is obtained by subtraction of both spectra, indicating the ligand protons that received saturation from the epitope. Thus, STD-NMR is applied to provide information about the binding epitope of the ligand to identify structure–activity relationships. Given the complexity of procyanidins, which exist as flexible rotamers that vary with the solvent and temperature, it is crucial to conduct NMR experiments under conditions close to the physiological environment (aqueous buffer, neutral pH, and 298 K) to accurately capture the interaction dynamics.^20,32^

To evaluate whether a stronger interaction results in a reduced α-amylase activity, the hydrolysis of the natural substrate starch in the presence of flavan-3-ols was determined by measuring the heat release using ITC. This novel approach allows for the monitoring of the rate of starch hydrolysis.

## 2 Materials and Methods

### 2.1 Chemicals and Materials

(+)-Catechin (CAT, ≥95% purity, lot 10742), procyanidin B1 (PC B1, ≥97% purity, lot 14872), procyanidin B2 (PC B2, ≥96% purity, lot 17603), and procyanidin C1 (PC C1, ≥97% purity, lot 15436) were obtained from Phytolab (Vestenbergsgreuth, Germany). (−)-Epicatechin (EC, ≥90% purity, lot BCBW4134), α-amylase (from porcine pancreas DFP-treated, type I-A, ≥1000 units/mg protein, lot SLCC7979), and starch from potato (lot 0000061135) were from Sigma-Aldrich (Taufkirchen, Germany). Disodium hydrogen phosphate and sodium dihydrogen phosphate (anhydrous, pure) and hydrochloric acid (HCl, 37%) were from Merck (Darmstadt, Germany). Deuterium oxide was purchased from Eurisotop (St.-Aubin Cedex, France), and deuterium chloride and sodium deuterium oxide were from Acros Organics (Basel, Switzerland). All chemicals were of analytical grade, and ultrapure water (ELGA PureLab Flex, Veolia Waters, Celle, Germany) was used throughout the experiments.

### 2.2 α-Amylase Preparation

To match the buffer for subsequent experiments, α-amylase was rebuffered into 0.1 M phosphate buffer containing 0.04 M NaCl at pH 7. For larger volumes (>0.2 mL) required for interaction studies by ITC, an Amicon Ultra-4 centrifugal filter device (Merck, Amicon Ultra-4, MWCO 50,000) was used (Hermle centrifuge Z326K, 7500 rcf, 293 K, 4 × 13 min). For STD-NMR experiments, small amounts of α-amylase (50–150 μL) were rebuffered in deuterated phosphate buffer using dialysis units (Thermo Fisher, Slide-A-Lyzer, MWCO 10,000). For enzyme kinetic measurements, α-amylase was diluted directly from its original bottle. Dilution was 1:1000 with 0.1 M phosphate buffer/0.04 M NaCl. Rebuffered protein was stored for max. 48 h at 277 K.

Protein concentration was determined in triplicate using a BMG Labtech SPECTROstar Nano (Ortenberg, Germany) with an LVis microplate for proteins at 298 K using a MW of 57,086 Da and an absorption coefficient ε of 2.436 L/g cm according to the UniProt Consortium 2021, ID P00690 (https://www.uniprot.org).

### 2.3 Flavan-3-ol Solutions

Flavan-3-ols were dissolved in 0.1 M phosphate buffer containing 0.04 M NaCl at pH 7. Final concentrations were determined by UV/Vis spectroscopy at 298 K (quartz glass cuvette, light path 10 mm, Hellma Analytics, SPECTROstar Nano, BMG Labtech) using the following absorption coefficients ε at 280 nm: CAT, 3340 [L/mol cm]; EC, 3470 [L/mol cm]; PC B1, 6800 [L/mol cm]; PC B2, 6800 [L/mol cm]; PC C1, 10,000 [L/mol cm] according to Kaeswurm et al.^33^ All ligand solutions were stored at 253 K.

### 2.4 Signal Assignment by NMR

Signal assignment was performed for the procyanidins (6.83 mM PC B1, 8.84 mM PC B2, and 2.87 mM PC C1 in methanol-*d*_4_ and D_2_O (1:1)) at a Bruker Avance III HD 700 NMR spectrometer equipped with a 5 mm QCI cryoprobe, ATM and a *z*-gradient, and a BCU I cryostat for sample temperature control (Bruker, Billerica, USA). Signal assignment was performed using a combination of 1D ^1^H (zg, zgpr, zggpw5, and noesygppr1d), ^1^H,^1^H-TOCSY, ^1^H,^1^H-NOESY, ^1^H,^1^H-ROESY, ^1^H,^13^C-HSQC, and ^1^H,^13^C-HMBC NMR spectra at 274 K. 2D TOCSY spectra were recorded with two different spinlock times (except if stated otherwise) of 100 and 20 ms, the latter for COSY-like information. 2D ROESY spectra were acquired with a spinlock time of 800 ms and 2D NOESY experiments with a mixing time of 800 ms. The spectra were acquired applying 50% nonuniform sampling (NUS) for all the data. The pulse sequences for the 1D and 2D NMR spectra and their respective acquisition parameters are available in the Supporting Information S1. Samples were stored at 253 K. To transfer signal assignment to STD-NMR conditions, spectra were also recorded for 7 mM PC B1, 7 mM PC B2, and 3 mM PC C1 in deuterated phosphate puffer at pH 7 (D_2_O/H_2_O, 90/10 v/v). Proton spectra were taken at 274, 298, and 310 K (274 K for 2D experiments). The data were acquired and processed with the software package TopSpin 3.2 (Bruker BioSpin).

### 2.5 Interaction Studies by Saturation Transfer Difference and Proton NMR

STD-NMR parameters were optimized based on the Bruker standard pulse sequence stddiffgp19.3 using a protein and ligand blank sample. The irradiation frequency (FQ2), saturation time (D20), saturation pulse selectivity (SPW9), and power of the saturation pulse (P42) were varied to achieve maximum selective protein saturation (S2). The best saturation of the α-amylase, with 60%, was obtained with FQ2 = 0.5 ppm, D20 = 3 s, SPW9 = 35 dB, and P42 = 50 ms. The pseudo-2D experiment allows simultaneous acquisition of the on-resonance spectrum (with selective protein saturation) and the off-resonance spectrum (without protein saturation). The subtraction of both FIDs yields the STD-NMR spectrum (S3). The off-resonance spectrum serves as a reference spectrum for the determination of ligand saturation by intensity comparison with the STD-NMR spectrum. The protein blank sample contained 10 μM α-amylase, and the ligand blank was acquired with 1.1 mM flavan-3-ol in 0.1 M phosphate buffer/0.04 M NaCl (D_2_O/H_2_O, 90/10, v/v). Interaction studies were performed with 10 μM α-amylase and 1 mM (ligand). The software package TopSpin 3.2 of Bruker BioSpin was used for data acquisition and processing.

^1^H NMR experiments were conducted to investigate the aggregation of PC C1 with α-amylase. First, 1 mM PC C1 (0.75 μmol) was measured, and then, α-amylase was added at concentrations of 10 and 20 μM. The relative decrease in intensity of PC C1 signals in the range of 6.866–7.040 ppm (E2′, H6′, B2′, B5′, E5′, E6′, and H5′) was used to calculate the proportion of PC C1 aggregated with α-amylase. Blank measurements were performed with 10 and 20 μM α-amylase dissolved in the same buffer without PC C1. Data were acquired using a Bruker Avance III HD 600 MHz NMR spectrometer equipped with a 5 mm BBFO probe (Smart Probe) with ATM and a *z*-gradient. Spectra were recorded at 275 K (2 °C) in 0.1 M phosphate buffer/0.04 M NaCl phosphate buffer, pH 7, in D_2_O:H_2_O (9:1). The pulse program zgesgp was used with a spectral width of 16 ppm, and all spectra were recorded with 512 scans, an acquisition time of 1.70 s, and a recycle delay (D1) of 1 s. Data were analyzed using TopSpin software (version 4.4.1, Bruker).

### 2.6 Changes in Protein Structure Studied by CD Spectroscopy

CD spectra were recorded in the range of 240–190 nm with a bandwidth of 1 nm on a Jasco J-815 CD spectrometer equipped with a Peltier unit from Jasco PTC-4238 and a temperature control (F250, Julabo). For CD experiments, 20 μL respective dilutions of the stock solutions for α-amylase, EC, and PC B2 or mixtures of α-amylase and flavan-3-ol, obtained from the ITC cell after interactions experiments (see Section 2.7; 90 μM α-amylase and 600 μM EC/PC B2), were diluted with 60 μL of 0.2 M KCl and filled bubble-free into a cuvette with a light path of 0.1 mm (Hellma Analytics). At 273 K, 40 independent spectra were accumulated in a continuous scanning mode and a speed of 100 nm/min without baseline correction. Data analysis was performed in Excel according to Kaeswurm et al.^34^

### 2.7 Interaction Studies by Isothermal Titration Calorimetry

Isothermal titration calorimetry was performed using an Affinity ITC LV with a cell volume of 182 μL (TA Instruments, Eschborn, Germany). Interaction experiments were recorded at 298 K with 0.1 mM rebuffered α-amylase in the sample cell and 4 mM flavan-3-ols in the injection syringe. For each ligand, experiments were performed with 25 injections of 3 μL and a spacing of 200 s between injections. Experiments were carried out in duplicates. Additional measurements were conducted in buffer to ensure that any mixing effects were negligible and that the observed heat development was solely attributed to the interaction studied (S4). In particular, at higher phenolic concentrations, endothermic peaks were observed, arising from the dilution effect.

The data analysis for the interaction studies was conducted using software NanoAnalyze, with manual integration of the peaks. To correct the data for noninteraction-related heats, the mean heat released during the final three injections was subtracted as a constant blank. This procedure is an effective estimation strategy that minimizes errors during the fitting process, provided that the protein is fully saturated.^35^ The data were fitted using the “independent” model. To prevent overparametrization, *n* was set to 1, while *K*_a_ and Δ*H* were maintained as variables, as recommended for low-affinity binding.^35,36^ The stability of the ligand at room temperature was ensured by monitoring the concentrations prior to and following the experiment (S5).

### 2.8 Continuous α-Amylase Activity Assay by Isothermal Titration Calorimetry

Without rebuffering, α-amylase was diluted each day with 0.1 M phosphate buffer/0.04 M NaCl to a final dilution of 1:1000 and stored at 4 °C. It was demonstrated that the heat development resulting from the difference in salt concentration between the protein and ligand solution was negligible (S6). The starch solution was prepared daily, according to the protocol recommended by Brodkorb et al. with some modifications.^37,38^

The starch suspension in 0.1 M phosphate buffer/0.04 M NaCl was heated for 5 min under vigorous stirring. After cooling to room temperature, the solution was diluted to a final mass concentration of 40.5 g/L (equivalent to 250 mM based on the molecular weight of the glucose monomers). The concentration of starch is typically expressed as a percentage by weight (%, w/v) due to the inherent polydispersity of the molecule, which is influenced by the source of the natural starch, the manufacturing process, and the preparation of the sample.^28,39^ However, the calculation of inhibition constants requires the use of molarities.

Control experiments were performed with 12 nM α-amylase in 0.1 M phosphate buffer/0.04 M NaCl placed in the sample cell at 310 K, and various volumes (1–10 μL) of starch solution were recurrently injected up to three times with an injection interval of 1600 s. It was elucidated that the optimal volume for reaching the maximal turnover velocity (*v*_max_) was 5 μL. Prior to measurement, α-amylase was incubated with the flavan-3-ols for 60 min at 277 K to ensure that adsorption equilibrium has been reached. This was compulsory as the time required for the ITC system to reach equilibrium and to start measurement varied considerably (10–25 min). To confirm α-amylase stability, controls without an inhibitor were performed at the beginning and the end of each day.

The release of heat during the conversion of starch was monitored, and calculations were performed using Microsoft Excel software. The detected thermal power is directly proportional to the enzymatic rate (*v*_0_) (eq 1 and 2).^40,41^
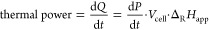
1
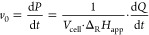
2

The heat rate (d*Q*/d*t*) is obtained as a function of time (*t*) and provides information about the conversion rate (ν). The reaction enthalpy (Δ_R_*H*_app_) is calculated by integrating the heat peak over its entire extent and dividing the result by the number of substrate molecules converted by the enzyme (eq 3). The substrate concentration at time *t* ([*S*]_*t*_) is calculated according to eq 4.
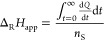
3
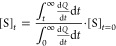
4

The Michaelis–Menten constant (*K*_m_) is defined as the substrate concentration at the time (*t*_0.5_) when the reaction rate reaches half-maximum (*v*_max_) (eq 5).
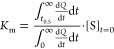
5

Thus, the thermogram provides all information to calculate inhibition constants. While competitive inhibition (*K*_ic_, eq 6) is based on the formation of an enzyme–inhibitor complex (EI), the uncompetitive inhibition (*K*_iu_, eq 7) refers to the binding of a ternary enzyme–substrate–inhibitor complex (ESI).
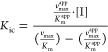
6
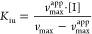
7

The maximal conversion rate *v*_max_ and the Michaelis–Menten constant *K*_m_ are calculated from the control experiments in the absence of an inhibitor. In the presence of an inhibitor [I], the apparent values of  and  have been determined.

The inhibitor concentration that is required to reduce the conversion rate to 50% (IC_50_) depends on the substrate concentration [S] and is calculated according to the Cheng–Prussoff equation (eq 8).^42^
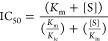
8

## Results

### 3.1 Interaction Studied by Isothermal Titration Calorimetry

**Figure 2:**
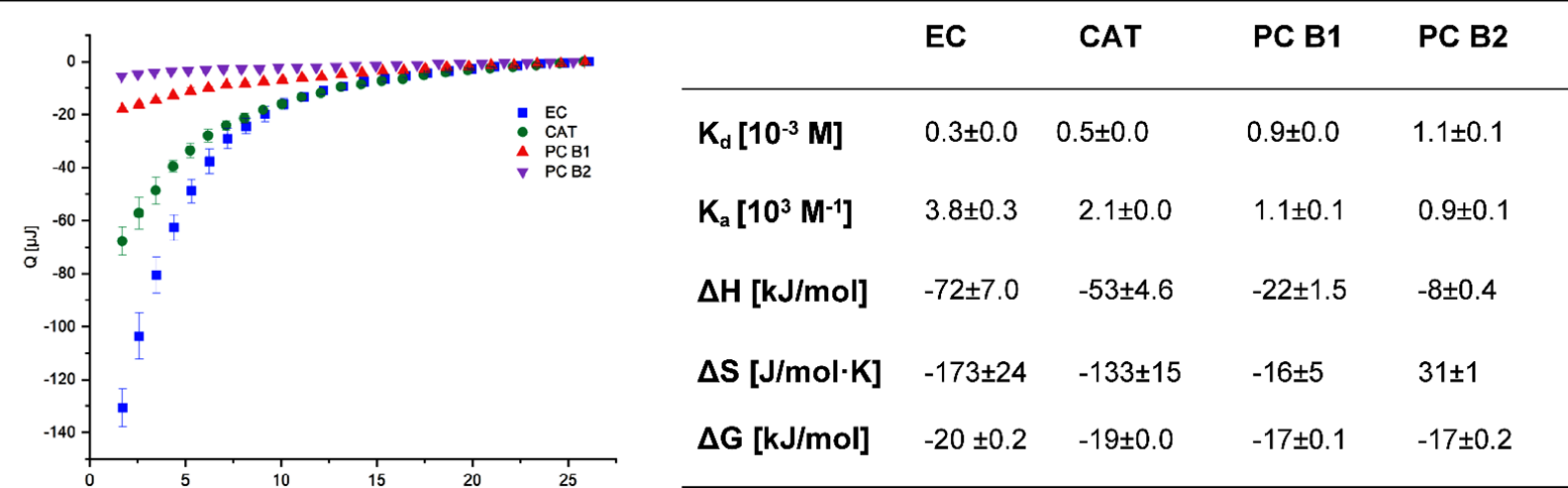
Converted thermogram (released heat vs number of injection) and thermodynamic parameters of the interaction of α-amylase with flavan-3-ols in phosphate buffer at 298 K.

**Figure 3:**
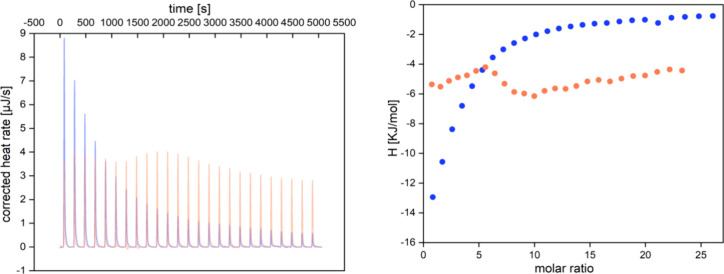
Overlay of the thermogram (left) and Wiseman plot (right) illustrating the interaction of 0.1 mM α-amylase with 4 mM EC (blue) and 4 mM PC C1 (orange).

The interaction of the flavan-3-ols with α-amylase was characterized by a low-affinity interaction, as indicated by the *K*_d_ values, which were in the millimolar range (Figure 2 and S7–S12). Nevertheless, for PC C1, the addition of the ligand resulted in fluctuating heat signals, which precluded the calculation of interaction parameters (Figure 3 and S11). ITC is a robust method to determine dissociation constants in the range from 100 μM to 1 nM, which are stronger than the binding affinities observed for all the flavan-3-ols studied.^29^ This weak interaction required to set stoichiometry *n* to 1 in order to fit the Wiseman plot, which might have an impact on the calculated thermodynamic parameters.

The heat release revealed distinct differences between the monomers and dimers but also between diastereomers (CAT–EC and PC B1–PC B2). During ligand addition, negative (exothermic) changes in heat were observed, which is energetically favorable and reveals the presence of noncovalent interactions.^43^ However, the release of heat was markedly lower for the dimers than for the monomers. In order to interpret this observation, it is compulsory to consider that the heat detected by ITC represents the overall energy changes of the experiment, including exothermic and endothermic proportions such as dilution, binding, solvation, and other energy variations due to conformational changes.^44^

Values for the Gibbs free energy Δ*G* were negative and in the same range, which indicates a spontaneous interaction for the monomers and dimers. The negative values for enthalpy and entropy are associated with the formation of hydrogen bonds between the protein and the ligand, solvent reorganization around the surface of the complex, and van der Waals interactions between the solvent and the protein and the solvent and the ligand.^29,44^ However, the impact of enthalpy and entropy on Gibbs free energy varied distinctly. While the exothermic enthalpies decreased in the following order: EC > CAT > PC B1 > PC B2, the impact of entropy increased. This compensation is most probably due to conformational restriction of the ligand (loss of freedom), when bound to the protein, but also, the release of the solvent from the binding area might be responsible for this effect.^29,45^ For PC B2, the enthalpic proportion was minimal and favorable positive Δ*S* were responsible for the spontaneous interaction.^43,46^ With PC C1, no interaction with α-amylase was observed, as illustrated in the thermogram and Wiseman plot (Figure 3). This is in contrast to all other flavan-3-ols, which exhibited a relatively consistent decline in heat with each successive addition of the ligand (S7–S10), a similar heat release was recorded with each injection (S11). The assumption that the heat is due to single precipitation events was supported by the observation of a turbid solution removed from the sample cell after the experiment.

### 3.2 Signal Assignment and Rotamer Ratios by 2D NMR

Studying interactions by STD-NMR requires a careful assignment of the ligands' proton resonances through 1D and 2D NMR experiments in advance. Usually, structure elucidation or proton signal assignment is performed under optimal conditions (e.g., methanol-*d*_4_) to obtain spectra with narrow line widths and reduced structure variations to limit signal overlaps. However, this is often far away from (*in vitro*) digestion conditions requiring aqueous media. In particular, for flavonoids, a pH-dependent equilibrium between charged and uncharged structures is well-known during the intestinal phase.^47^ Furthermore, due to the structural flexibility of procyanidins, they exist as different rotamers, depending on the solvent and temperature. Therefore, signal assignment under optimal conditions must be transferred to settings that are closer to the intestinal phase, like aqueous phosphate buffer at 310 and 298 K, which have been used for STD-NMR experiments. Besides elucidating the binding epitope, rotamers might interact differently with α-amylase, thus knowledge about the rotamer ratios is essential for a correct data interpretation.

**Figure 4:**
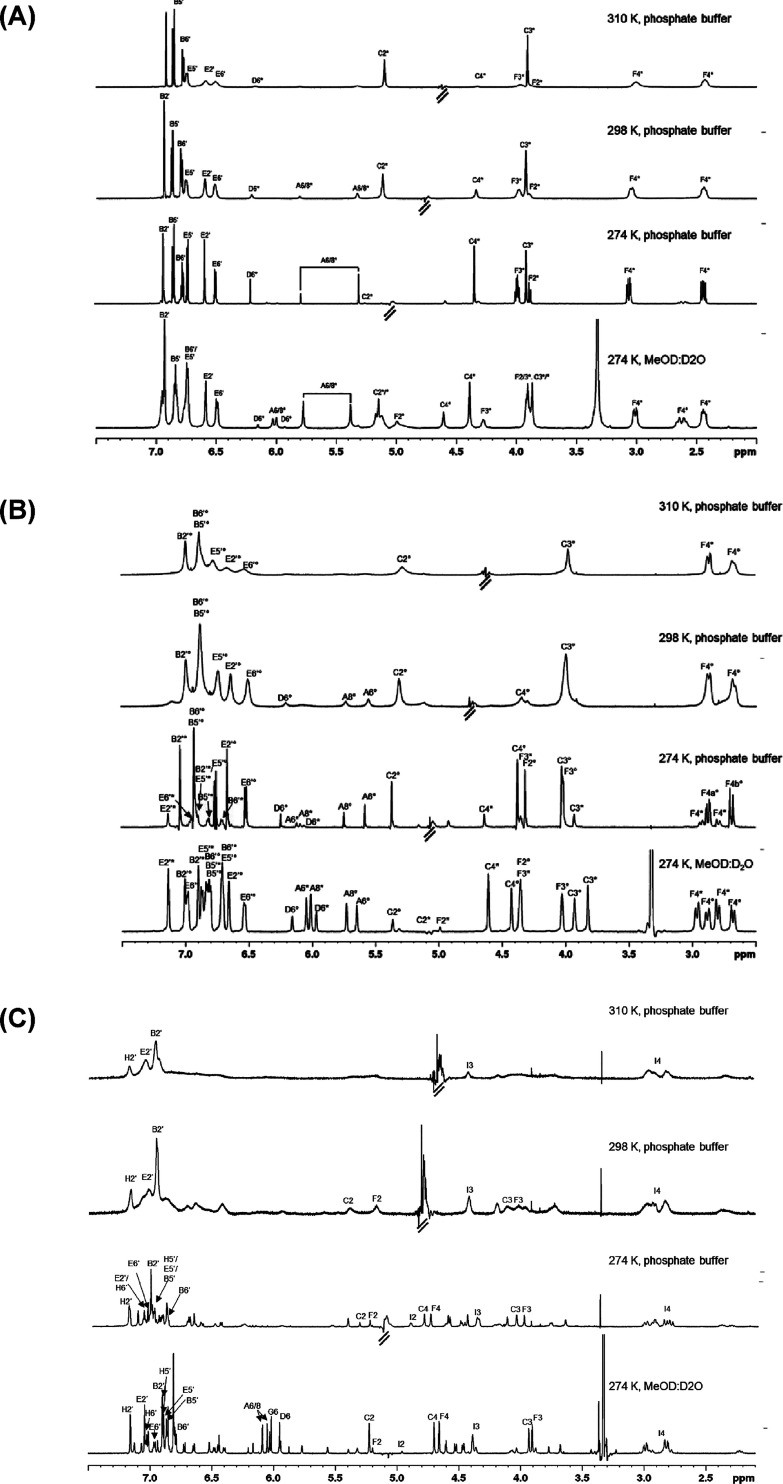
Stacked proton spectra (noesygppr1d) of (A) PC B1, (B) PC B2, and (C) PC C1 in methanol-*d*_4_:D_2_O (1:1) at 274 K and in 0.1 M PBS/D_2_O at 274, 298, and 310 K at 700 MHz.

The assignment of the protons and ^13^C resonances of the monomers EC and CAT is available from the literature.^48,49^ For the procyanidins, a set of 1D an 2D NMR spectra were acquired to assign the individual proton resonances of the main rotamers (S13–S42). Signal assignment was first performed in the solvent methanol-*d*_4_:D_2_O (1:1) at 274 K and then transferred to the spectra recorded at STD-NMR settings, which are closer to physiological conditions. For PC B1, PC B2, and PC C1, the aromatic proton shifts were divided into two distinct regions attributed to the differences in aromaticity and a third aliphatic region (Figure 4 and S13, S25, and S37).

More signals than proton environments were detected in the spectra of all procyanidins, indicating the presence of rotamers (Figure 4). For the dimers, two rotamers were observed, which have been reported previously.^32^ To distinguish between both rotamers for PC B1 and PC B2, the signals were labeled with a star (*) for the major and a circle (°) for the minor rotamer (Figure 4A,B). According to previous findings for the dimers in H_2_O/D_2_O (9:1) solutions, these rotamers are referred to compact and extended procyanidin structures depending on the dihedral angle of the interflavanoid bond (C4 to D8).^19,32^

The spectral quality for PC B1 was limited in methanol-*d*_4_:D_2_O (1:1) at 274 K, and the rotamer ratio could not be estimated for PC B1. In contrast, for PC B2, the signals of both rotamers were assigned (S26), at least for the heterocyclic rings C and F, to express the ratio between both rotamers to 55:45 (S32). The spectrum of PC C1 is considerably more complex due to up to eight different rotamers.^50^ In methanol-*d*_4_:D_2_O (1:1), at least three different rotamers were present making it very difficult to allocate the signals. Hence, only the signals from the major rotamer (*) were assigned for PC C1 (S38).

Interaction experiments by STD-NMR are preferably performed under aqueous conditions at room temperature or even at 310 K. Therefore, signal assignment was transferred to procyanidins dissolved in 0.1 M deuterated phosphate buffer and measured at relevant temperatures (Figure 4A–C). Correct identifications of signals were confirmed with ^1^H–^1^H TOCSY experiments (S22, S33, and S41). While it was challenging for PC B1 to distinguish between the signals of both rotamers in the methanolic solvent, this problem disappeared in phosphate buffer due to the existence of only one predominant rotamer (*) (Figure 4A). Furthermore, under these conditions, the resolution at 274 K was sufficient to identify coupling constants (S24). As expected, line broadening was observed at elevated temperatures, except for the signals of the B and C ring. At 310 K, the signal for C4 shifts in the region of water suppression and differentiation between the protons A8 and A6 was not possible.

For PC B2, two rotamers were identified in phosphate buffer analogous to aqueous methanol (Figure 4B). However, the major rotamer (characterized by higher integrals) in aqueous methanol converted to the minor rotamer in the phosphate buffer, resulting in an inversion of the integral ratio from 55:45 ((*):(°)) to 25:75 ((*):(°)) (Table 1 and S32 and S36). In order to keep the identifiers, the (°) rotamer indicates in buffer the component with higher integrals. Significant line broadening for all signals was observed with increasing temperature, and only the distinct F4 protons and the aromatic protons of ring B and E of the major (°) rotamer were assigned.

**Table 1:** Rotamer Ratios ((*):(°)) in % for PC B1 and PC B2 Depending on the Solvent

	**PC B1**	**PC B2**
methanol-*d*_4_:D_2_O	67:33^a^	55:45
phosphate buffer	100:0 (tr)	25:75

a Calculated from the protons F4; tr, traces of the minor signals were observed. Calculation according to the integrals provided in S21, S23, S32, and S36.

The challenging identification of the major signals for PC C1 in aqueous methanol was further complicated in phosphate buffer (Figure 4C and S41 and S42). A similar pattern for the aliphatic peaks was observed; however, signal intensities for protons belonging to ring A, D, and G were significantly reduced. In contrast to PC B1 and B2, a much more prominent line broadening occurred for the aromatic rings B, E, and H. This phenomenon was intensified at higher temperatures, and therefore, at 310 K, only single peaks could be assigned to certain protons.

### 3.3 Saturation Transfer from α-Amylase to the Flavan-3-ols and Aggregate Formation

**Figure 5:**
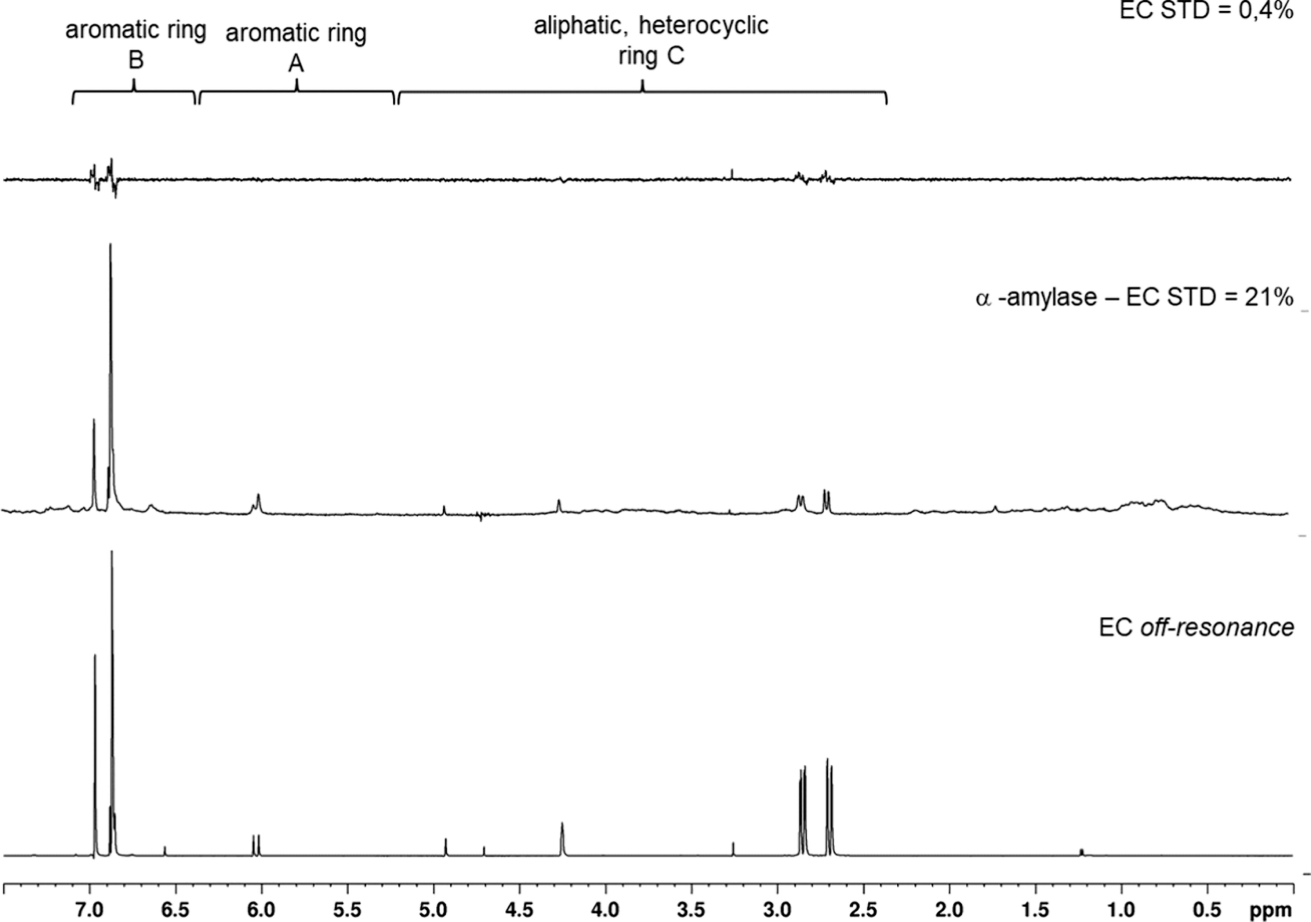
Stacked STD spectra of 1 mM EC in 0.1 M PBS/0.04 M NaCl (D_2_O/H_2_O, 90/10 v/v) in the absence (top) and the presence of 10 μM α-amylase (middle). Off-resonance spectrum of 1 mM EC at 700 MHz at 298 K (bottom). Saturation for the STD spectra was determined by scaling the aromatic B ring signals and is indicated in percentage.

To identify the binding epitope of the individual flavan-3-ols to the protein, it must be ensured that the ligands do not experience any saturation from the saturation pulse, but from protein saturation transfer only. Saturation of the ligands in blank samples without protein was tested and determined to be below 1%, except for CAT with around 2% (Figure 5 top spectrum). Distinct STD-NMR signals were detected, clearly indicating molecular interactions between α-amylase and all ligands. Transfer of saturation from α-amylase to the ligands was in the range of 9–21% for the aromatic and 3–11% for the aliphatic protons (Table 2 and S43–S46). It is noteworthy that the saturation of the monomer EC (Figure 5) is approximately twice that of the diastereomer CAT, which suggests a lower dissociation rate of the former. The ratios between saturation of aromatic and aliphatic protons were 79:21 for the monomers and significantly lower with 59:41 for the procyanidin di- and trimers.

**Table 2:** Saturation of Flavan-3-ols during Interaction with α-Amylase Differentiated between Scaling to the Aromatic (arom.) and Aliphatic (aliph.) Protons and Their Respective Ratio

		**blank**		**protein–ligand**		**ratio**		**average**^b^	
		arom.	aliph.	arom.^a^	aliph.^a^	arom.	aliph.	arom.	aliph.
EC	0.4	21	6	21	6	79	21		
CAT	2	13	5	11	3	79	21	79 ± 0	21 ± 0
PC B1	0.3	14	11	14	11	56	44		
PC B2	0.3	9	6	9	6	60	40		
PC C1	0.3	11	7	11	7	61	39	59 ± 3	41 ± 3

a Corrected by a blank.

b Average according to monomer and di-/trimer groups. As the signals of phloroglucinol rings A, D, and G were almost undetectable in the STD spectra, only the aromatic signals of the catechol rings B, E, and H could be considered.

However, for the trimer, precipitation in the NMR tube was observed after conducting the STD experiment. Nevertheless, an STD spectrum was obtained, suggesting that some α-amylase was still interacting with the trimer in solution (S46). To estimate the PC C1 concentration in solution after precipitation, the difference between the integrals of aromatic protons in the off-resonance spectra from the STD spectra and the pure ligand sample (blank) was calculated, suggesting a decrease by roughly 65% and a PC C1 concentration of 0.35 mM.

**Figure 6:**
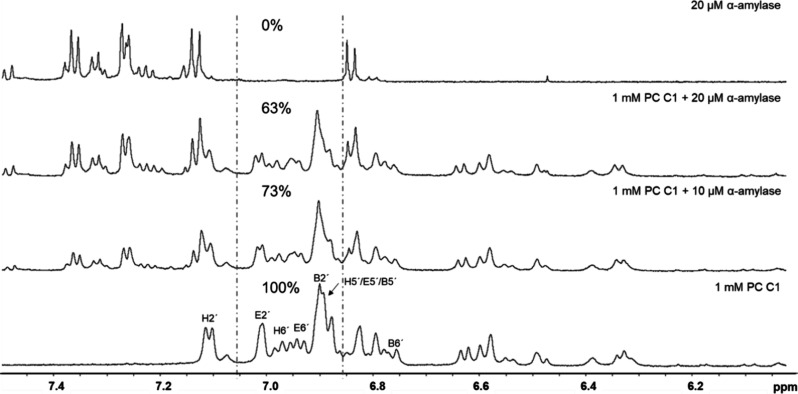
Assessed proportion of PC C1 aggregated with α-amylase. The spectral range to calculate the decrease in PC C1 signal intensities (%) following α-amylase addition is indicated in the proton spectra (zgesgp) at 275 K in 0.1 M phosphate buffer (D_2_O:H_2_O 9:1, 0.04 M NaCl, pH 7).

The formation of PC C1−α-amylase aggregates was monitored by proton NMR (Figure 6 and S47 and S48). PC C1 (1 mM) signal intensities were reduced to 73% and 63% by the addition of 10 and 20 μM α-amylase, respectively. This indicates adsorptions of 0.20 and 0.28 μmol PC C1 on the surface of α-amylase. The ratio of PC C1 to α-amylase is reduced from 26 (10 μM α-amylase) to 18 (20 μM α-amylase), which might be interpreted, which might be interpreted as an increased formation of higher amylase aggregates, mediated by the PC C1.

### 3.4 Change in Secondary Structure of α-Amylase

**Figure 7:**
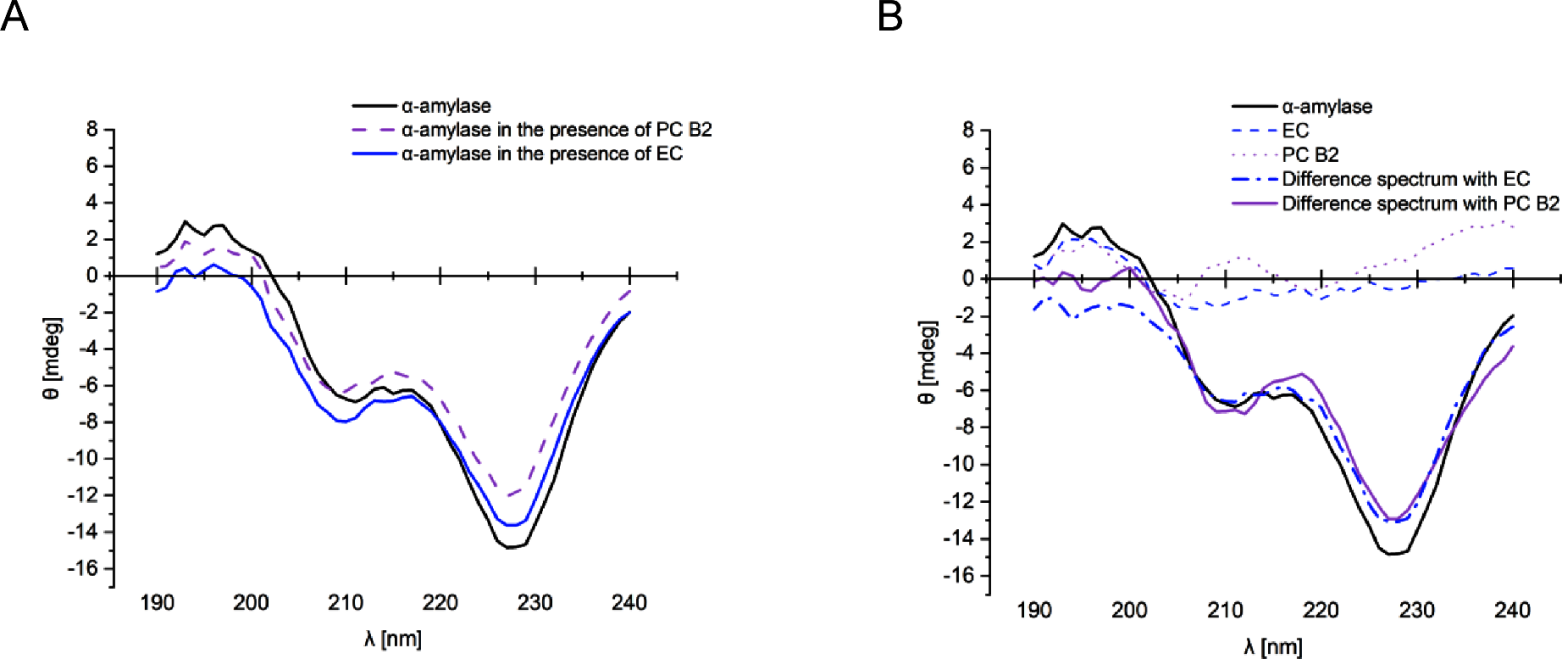
Original CD spectra of 90 μM α-amylase and α-amylase in the presence of 600 μM EC and PC B2 (A). CD difference spectra with 90 μM α-amylase and 600 μM EC and PC B2 (B).

To obtain information about the impact of the interaction of flavan-3-ols on the secondary structure of α-amylase, CD spectra were recorded. No significant impact on the secondary structure of amylase was observed with EC and PC B2 (Figure 7 and S49). EC itself exhibited minimal CD activity around λ = 195 nm, and the spectrum of the mixture was comparable to the spectra of pure α-amylase. PC B2 exhibited enhanced CD activity, and the spectrum of the mixture was a summation of the CD activity from α-amylase and PC B2. Calculating the ratio between secondary structure elements according to Provencher and Glöckner^51^ revealed some alterations of the values for the α-helix and β-turn (S49). However, the changes were interpreted as artifacts coming from the CD activity of the ligands around 190 nm, the wavelength range with a relevant impact on the values for the α-helix and β-turn (Figure 7B). Acquisition of a CD spectrum in the presence of PC C1 was impossible due to precipitation (data not shown).

### 3.5 Inhibition of Starch Conversion by Flavan-3-ols

**Figure 8:**
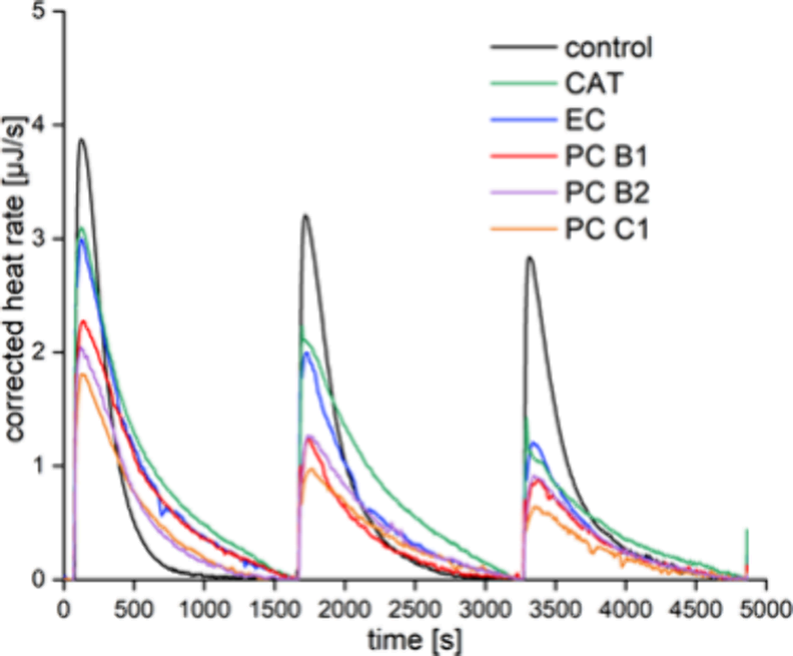
Thermogram of recurrent single injection experiments of starch conversion (7 mM/injection) titrated into α-amylase (12 nM) incubated with 100 μM flavan-3-ol at 310 K (left).

The kinetics of α-amylase inhibition was investigated by ITC, monitoring the heat release during starch conversion. The starch in the ITC syringe was titrated up to three times into α-amylase incubated with the inhibitor. Even in the control, without any inhibitor, *K*_m_ increased slightly and *v*_max_ decreased notably throughout the three injections (Table 3, Figure 8, and S50 and S51). This indicates product inhibition, for which *K*_m_ and *v*_max_ suggest a mixed inhibition. Calculation of the competitive and uncompetitive proportion is not feasible due to the unidentified products and thus their concentrations, which act as inhibitors. As a result, inhibition experiments with flavan-3-ols were generally affected by product inhibition. In order to minimize this interference, only the data obtained from the initial injection have been used to evaluate the inhibitory impact of the flavan-3-ols (Table 4). The effect of product inhibition is less important under *in vivo* conditions where the malto-oligosaccharides are further hydrolyzed by brush border enzymes to form glucose, which is absorbed by the intestinal epithelial cells.

**Table 3:** *v*_max_ and *K*_m_ for Recurrent Single Injection Experiments of Starch Titration into α-Amylase at 310 K^a^

	**inj. 1** mean ± SD	**inj. 2** mean ± SD	**inj. 3** mean ± SD	**difference between injections 1–3**
*K*_m_ [mM]	2.33 ± 0.10	2.79 ± 0.09	2.87 ± 0.35	+23%
*v*_max_ [mmol min^–1^ L^–1^]	1.71 ± 0.05	1.43 ± 0.05	1.23 ± 0.14	–28%

a *n* = 3 (concentrations in the sample cell: 12 nM α-amylase, 7 mM starch/5 μL injection volume).

**Table 4:** Inhibition of α-Amylase with 100 μM Flavan-3-ols in Phosphate Buffer at 310 K^c^

**inhibitor**	*v*_**max**_**[%]**	**(d***Q***/d***t***)_**max**_**[%]****	**Δ**_**R**_***H***_**app**_ [J/mol]	***K***_**ic**_**[μM]**	***K***_**iu**_**[μM]**	**α(*K***_**ic**_**/*K***_**iu**_**)**	**IC**_**50**_**[μM]**^a^
control	100	100	727 ± 103				
EC	78 ± 4	82 ± 8	816 ± 53	253 ± 47	385 ± 82	0.7 ± 0.1	385 ± 82
CAT	79 ± 6	80 ± 13	677 ± 140	356 ± 209	431 ± 217	0.8 ± 0.4	431 ± 217
PC B1	66 ± 9	66 ± 14	653 ± 96	155 ± 79	219 ± 91	0.7 ± 0.2	219 ± 91
PC B2	62 ± 4	56 ± 4	856 ± 33	177 ± 39	173 ± 26	1.0 ± 0.1	173 ± 26
PC C1	50 ± 4	44 ± 4	599 ± 33	94 ± 26	103 ± 16	0.9 ± 0.1	103 ± 16
PC C1^b^	(98 ± 21)	23 ± 0	170 ± 42				

a Calculated according to the Cheng–Prussoff equation (eq 8) for a 1 M substrate.

b Data obtained with PC C1 concentrations of ∼105 μM.

c Mean values ± SD (*n* = 4).

A reversible inhibition is defined by a constant reaction enthalpy (Δ_R_*H*_app_). The release of a comparable quantity of heat indicates a corresponding quantity of product formation, even at a reduced hydrolysis rate. Thus, inhibitory strength is most effectively exemplified by the decline in *v*_max_ and (d*Q*/d*t*)_max_, and the ratio of *v*_max_ and (d*Q*/d*t*)_max_ to the value observed in the control is equivalent. The occurrence of irreversible inhibition results in a reduction of Δ_R_*H*_app_, which consequently indicates that (d*Q*/d*t*)_max_ is a better representation of the inhibition than *v*_max_, which is dependent on Δ_R_*H*_app_ (eq 2). Although there are some notable deviations in Δ_R_*H*_app_, it is evident that for monomers and dimers, inhibition is due to reversible interactions (Table 4). The decline in Δ_R_*H*_app_ in the presence of 100 μM PC C1 indicated an irreversible proportion, which was confirmed by repeating the experiments with a slightly higher concentration (S52). Additionally, these data illustrate the limitations of using *v*_max_ as an indicator in the context of irreversible inhibition.

It was apparent that the inhibitory effect of both procyanidin dimers and the trimer exceeded that of the monomers. The reduction in (d*Q*/d*t*)_max_ relative to the control was significantly greater in the presence of dimers and trimers compared to monomers. This trend was further supported by the calculated inhibitory constants, *K*_ic_ for the competitive and *K*_iu_ for the uncompetitive inhibition, derived from the thermogram (eqs 6 and 7). A lower value indicates stronger inhibition. Additionally, the ratio of , known as the α value, implicated the mechanism of inhibition. An α-value <1 describes a mixed inhibition with a higher proportion of competitive inhibition, where the flavan-3-ols interact more with the enzyme than binding to the enzyme–substrate complex. The IC_50_ value, which represents the concentration of the inhibitor, required to reduce the enzyme activity by 50%, approaches the *K*_iu_ value for high substrate concentrations, further demonstrating that the inhibitory impacts of the dimers and trimer were stronger than those of monomers. Notably, PC B2 showed a slightly lower IC_50_ Value compared to PC B1, despite both being diastereomers. The difference in the inhibition strength between the dimers PC B1 and PC B2 is due to a higher uncompetitive proportion of PC B2, which binds more effectively to the enzyme–substrate complex.

## 4 Discussion

### 4.1 ITC and STD-NMR Reveal Weak and Unspecific Reversible Interactions of α-Amylase with Monomeric and Dimeric Flavan-3-ols

The data obtained from both methods indicate that the interaction of monomeric and di- and trimeric flavan-3-ols with α-amylase is limited. However, they also highlight a notable distinction between the monomeric and dimeric diastereomers. Through ITC and STD-NMR, it was determined that EC exhibited a stronger interaction with α-amylase compared to CAT and despite a significantly weaker affinity for the dimers, PC B1 interacts more than PC B2.

Studies investigating the interaction of flavan-3-ols with proteins using ITC and STD-NMR are scarce; however, Sun et al. identified EC as a weak binder for α-amylase by ITC.^28^ The even lower heat release during the experiment, which precluded the fitting process and the acquisition of thermodynamic parameters, was likely due to an α-amylase concentration of 20 μM in the sample cell, which was considerably lower than the 100 μM used in the present study. For high-affinity bindings, the curve in the Wiseman plot would exhibit a sigmoidal shape with a distinct inflection point. Conversely, for weak bindings, the binding isotherm deviates from this shape, assuming a hyperbolic form.^44^ Due to the absence of a clear inflection point and to avoid an overparametrization of Δ*H* and *K*_d_ during the fitting process, the stoichiometric parameter *n* was fixed to 1, which has been suggested by several experts in the field.^35,36,29,52^ This contrasts with the assumed unspecific interaction, where *n* is most probably greater than 1 and also dependent on the ligand size. Thus, this prohibits an overinterpretation of the *K*_d_ values. Furthermore, flavan-3-ols, as low-affinity binders, require a considerable number of injections to achieve a constant baseline at the end of the titration, which involves additional uncertainty due to the expansion of the interaction volume. An increase in ligand and protein concentration might help to overcome this issue; however, the insolubility of the ligand itself or precipitation and instability of the protein were limiting factors.

Negative enthalpy Δ*H* indicates an interaction primarily driven by hydrogen bonds and van der Waals forces. Hydrophobic interactions, which might occur between the aromatic or aliphatic side chains of proteins and the aromatic rings of phenolic compounds, would contribute to a positive change in entropy.^43,53^ The slight decrease in affinity from EC to PC B2 coincides with a minor decrease in the Gibbs free energy Δ*G* and a substantial reduction in the (negative) enthalpy. Thus, entropy, indicating an increase in hydrophobic interactions, predominates. STD-NMR also revealed PC B2 as the weakest binder.

The disparate thermodynamic behavior of PC B1 and PC B2, in particular the positive entropy values observed for PC B2, might be attributed to the different stiffness of their structures, indicated by the coexistence of two rotamers for PC B2. The entropy can be interpreted as the measure of the disorder within a system.^43^ The total change in entropy of protein–ligand binding comprises the entropy change due to solvent release from the binding interface (Δ*S*_solv_), the conformational freedom of both the protein and ligand upon binding (Δ*S*_conf_), and the loss of rotational and translational degrees of freedom of the protein and ligand upon complexation (Δ*S*_complex_). While the solvent release, which might also result from aggregation, is characterized by a large positive Δ*S* value, the formation of a protein–ligand complex decreases the particle number in solution and thus contributes unfavorable (negatively) to the binding entropy. It can thus be postulated that the solvent release may serve as a weak driving force for the interaction of α-amylase with PC B2.^44^ The monomers EC and CAT are per se more rigid due to the lack of interflavan bonding. The higher negative Δ*H* values, which are attributed to the formation of hydrogen bonds and van der Waals forces, indicate that binding is less sterically hindered compared to the dimers.^43^

The substantial entropy increases for the dimers, indicating that denaturation of the α-amylase is less pronounced. Denaturation is an entropically unfavorable process since hydrophobic groups, which were previously localized inside the protein, become exposed to the polar solvent.^46^ This exposure would provoke the organization of the polar solvent molecules, which is known as solvation cages, around the hydrophobic groups (hydrophobic effect). The fact that denaturation is less important is supported by CD spectroscopy. The addition of EC and PC B2 did not induce significant changes in the secondary structure of α-amylase. This finding is in contradiction with the study by Dai et al., which observed an increase in the proportion of the α-helix and β-sheet in the presence of PC B2.^54^ However, unfortunately, a PC B2 blank spectrum was not provided in their study. Based on our data (Figure 7), we would propose that the change in the α-amylase spectrum in the presence of PC B2 is the sum of both individual spectra presented. Cai et al. identified a decrease in the proportion of the β-sheet and an increase in the α-turn and random coil with an increasing concentration of PC B2, but which might also be due to the additional impact of a ligand blank on the spectra as illustrated by our calculations (S49).^55^

So far, the most common techniques used to determine the interaction of α-amylase with phenolic structures are fluorescence quenching, UV spectroscopy, and circular dichroism (CD) spectroscopy. Cai et al. determined a *K*_a_ value of 872 M^–1^ with a mixture of eight procyanidin trimers by fluorescence quenching.^55^ In our study, for the dimers PC B1 and PC B2, the reported values were higher with 1100 and 900 M^–1^, respectively. However, for PC C1, the values could not be determined, likely due to precipitation events. The accurate determination of ligand and protein concentrations is a prerequisite for the correct calculation of binding affinities.^29^ In particular, inadequate quantification of the ligand concentration is the most important source of error, which is directly translated to the thermodynamic parameters obtained from the fitting procedures. For proteins, quantification by UV spectroscopy does not indicate correct folding. Consequently, despite the precise determination of concentration, deviations in *K*_d_ and Δ*H* may occur due to partial denaturation of the protein. The errors in Δ*H* are multiplied by the van’t Hoff equation for Δ*S*.

### 4.2 The Differences in the Configuration and the Spatial Dimension Affect the Interaction

The comprehensive NMR investigations provided valuable insights into the relevant structures under the conditions used for the interaction experiments. Notably, for procyanidin dimers, a clear shift in rotamer ratios was observed, highlighting the significance of assigning signals and elucidating structures in the same solvent used for interaction studies. For PC B1, only one dominant rotamer was identified in a phosphate buffer, whereas for PC B2, two rotamers were present in a 25:75 ratio. The rotation of the dimers occurs between the interflavan link (C4–D8), while rotation around the single bond between C2 in the heterocyclic C ring and the carbon 1′ in the B ring and similar between the F and E ring is very fast.

**Figure 9:**
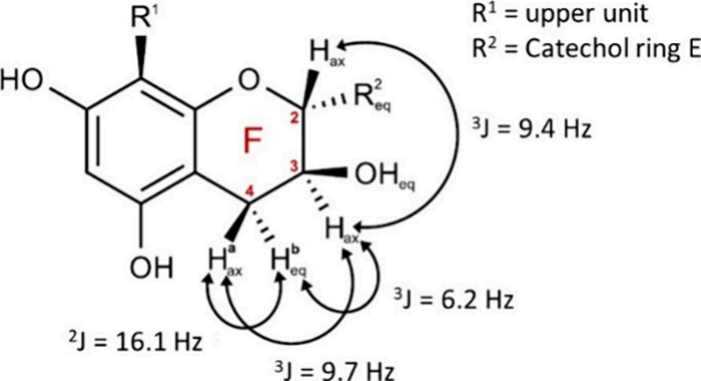
Stereochemical assignment of the PC B1 heterocyclic F ring from the coupling constant in phosphate buffer at 274 K.

The substantial disparity between PC B1 and PC B2 was unexpected, given that both structures are analogous except for the one stereochemical positioning of the hydroxyl group on carbon F3. The coupling constants for PC B1 in phosphate buffer (Figure 9 and S24) were found to be very small for protons at carbons C2, C3, and C4 in the upper unit, which suggests that these protons have a dihedral angle of approximately 90° (Karplus relation 2 Hz). The determination of significantly larger coupling constants for the lower monomer unit indicated some distortion of the chair arrangement. The coupling constant of 9.4 Hz between the protons of F2 and F3 suggests a diaxial conformation with a dihedral angle of approximately 180° referred to the protons.

The differences in the rotamer ratio between aqueous and alcoholic solvents have been already reported by Tarascou et al. in their study of procyanidins in a simulated wine medium.^32^ They observed that the compact form with an equatorial conformation of the heterocyclic rings is largely preferred in water or in a wine-like medium for PC B1, with slightly higher amounts of the minor extended rotamer in hydro-alcoholic (8%) than in aqueous media (5%). In our study with phosphate buffer, only traces of the minor rotamer for PC B1 were detected; however, at 50% methanol, the proportion of the minor rotamer increased to 33%. For PC B2, a significantly higher proportion of the extended rotamer was observed by Tarascou et al., with values of 45% in water and 47% in hydro-alcoholic media. This is in accordance with our data obtained for PC B2. However, in our study, only 25% of the minor rotamer was detected in phosphate buffer, which is most probably the extended rotamer. Interestingly, the proportion increased to 55% in aqueous methanol (1:1), indicating that PC B2 is a more flexible molecule than PC B1. Considering the findings presented by Tarascou et al., it can be postulated that, at least for PC B1, the compact rotamer is involved in the interaction with α-amylase.

Investigating the rotamer ratios for PC B1 (EPI–CAT), PC B2 (EPI–EPI), PC B3 (CAT–CAT), and PC B4 (CAT–EPI) in detail, Tarascou et al. found that the amount of the extended rotamer is dependent on the stereochemistry at carbon 3 of the lower unit. The compact form with an equatorial conformation of the heterocyclic ring was observed to be the predominant preference for the CAT in the lower unit. The impact of the solvent on the procyanidin rotamer distribution appears to be most significant in aqueous and alcoholic media and mixtures thereof. While minor rotamers were observed in our study for PC C1, Shoji et al. and Esatbeyoglu et al. identified only one rotamer in CD_3_OD and acetone-*d*_6_, respectively.^56,19^

The NMR data substantiate that the differences in enthalpy and entropy between PC B1 and PC B2 determined by ITC are most likely due to the present rotamers. While PC B1 exhibits a single rotamer in phosphate buffer, PC B2 displays enhanced flexibility, leading to the formation of two distinct conformers with notable differences in their spatial dimensions and most probably also in their binding affinity. This is supported by the proton spectra of the procyanidins at 310 K, mimicking the conditions for interaction and inhibition experiments. For PC B2 and PC C1, a notable line broadening indicates a shift from a low exchange rate to medium interchange. In contrast, for PC B1, the signals of the upper unit remained sharp indicating a low exchange, and only the signals for the lower unit decreased due to line broadening.

STD-NMR experiments are optimal for interactions with *K*_d_ values ranging between 10^–3^ and 10^–8^ [M].^31^ This exchange rate should present clear STD spectra, while in contrast to weak or strong ligand binding, a sufficient number of saturated ligands are present in solution. The saturation transferred from α-amylase to the investigated ligands CAT, PC B1, PC B2, and PC C1 ranged between 9 and 21%. Notably, EC exhibited the greatest saturation, which correlates to the strongest interactions among all flavan-3-ols, as determined by ITC interaction experiments. The ligands undergo saturation only when bound to the protein. After dissociation from the protein, the saturated state is accumulated in solution due to limited relaxation. Therefore, the ligand saturation [%] depends strongly on reversible binding kinetics and the excess of the ligands over the protein.

There is a lack of literature regarding interaction experiments of α-amylase and procyanidins by STD-NMR. In a previous study, Kaeswurm et al. performed STD-NMR with chlorogenic acid and phlorizin, observing generally a higher proportion of saturation for the aromatic protons.^57^ However, no monomeric flavan-3-ols or procyanidins were investigated. In the present study, it is remarkable that aliphatic protons in procyanidin di- and trimers receive a significant higher saturation (59:41, aromatic:aliphatic) than in the monomers (79:21, aromatic:aliphatic). It should be noted that aromatic protons generally have longer relaxation times compared to aliphatic protons; however, the difference in saturation was substantial, indicating a binding via the aromatic protons. There are some options regarding the binding arrangement:

1. The protons of the catechol rings might interact with the hydroxyl groups via hydrogen bonds and π-stacking of the aromatic rings. The short residence time of the protein–ligand complex limits spin diffusion throughout the ligand and keeps saturation exclusively on the binding protons. The enhanced saturation observed for the aliphatic protons of the di- and trimers may be a result of spin diffusion, which depends on the lifetime of the protein–ligand complex.
2. Another explanation would suggest binding of all aromatic protons (including the phloroglucinol protons), but relaxation time is significantly lower for phloroglucinol protons than for catechol protons, which benefit from the free rotation between the heterocycle and the catechol ring.
3. Moreover, the signals of the phloroglucinol protons may be undetectable due to hydrogen–deuterium exchange. The interaction of PC B4 (CAT-EPI) with mucin was investigated by Brandão et al.^58^ They identified the phloroglucinol protons as the main interacting protons, despite also acquiring the spectra in D_2_O, which does not support the theory of hydrogen–deuterium exchange. It can thus be concluded that the binding of CAT, EPI, PC B1, PC B2, and PC C1 to α-amylase may occur exclusively via the catechol rings.

In their investigation into the interaction of PC B3 with the digestive enzyme trypsin, Gonçalves et al. observed that binding predominantly occurred via aromatic protons, a finding that aligns with our observations regarding α-amylase.^59^ Additionally, Soares et al. identified high affinities between PC B3 and C2, as well as other flavanols, and proline-rich salivary proteins.^60^ However, they concluded that the number of hydroxy groups had a significant impact on binding. For the flavan-3-ols investigated, the dimers and trimer are considerably more sterically demanding and thus the positions of the aromatic rings might be suboptimal for interaction and result in a reduced saturation of aromatic protons as compared to the monomers. On the other side, procyanidins may exhibit slower binding kinetics due to the potential rearrangement of the structure around the interflavan bond to optimize binding. To gain more detailed insight into the association and dissociation rate constants, surface plasmon resonance experiments could be conducted. This real-time method would also provide information on the stoichiometry of binding, since the angular change in total reflectance measured here is proportional to the bound mass.

### 4.3 Impact of the Stereocenters/Rotamers and the Degree of Polymerization of Flavan-3-ols on α-Amylase Inhibition

**Figure 10:**
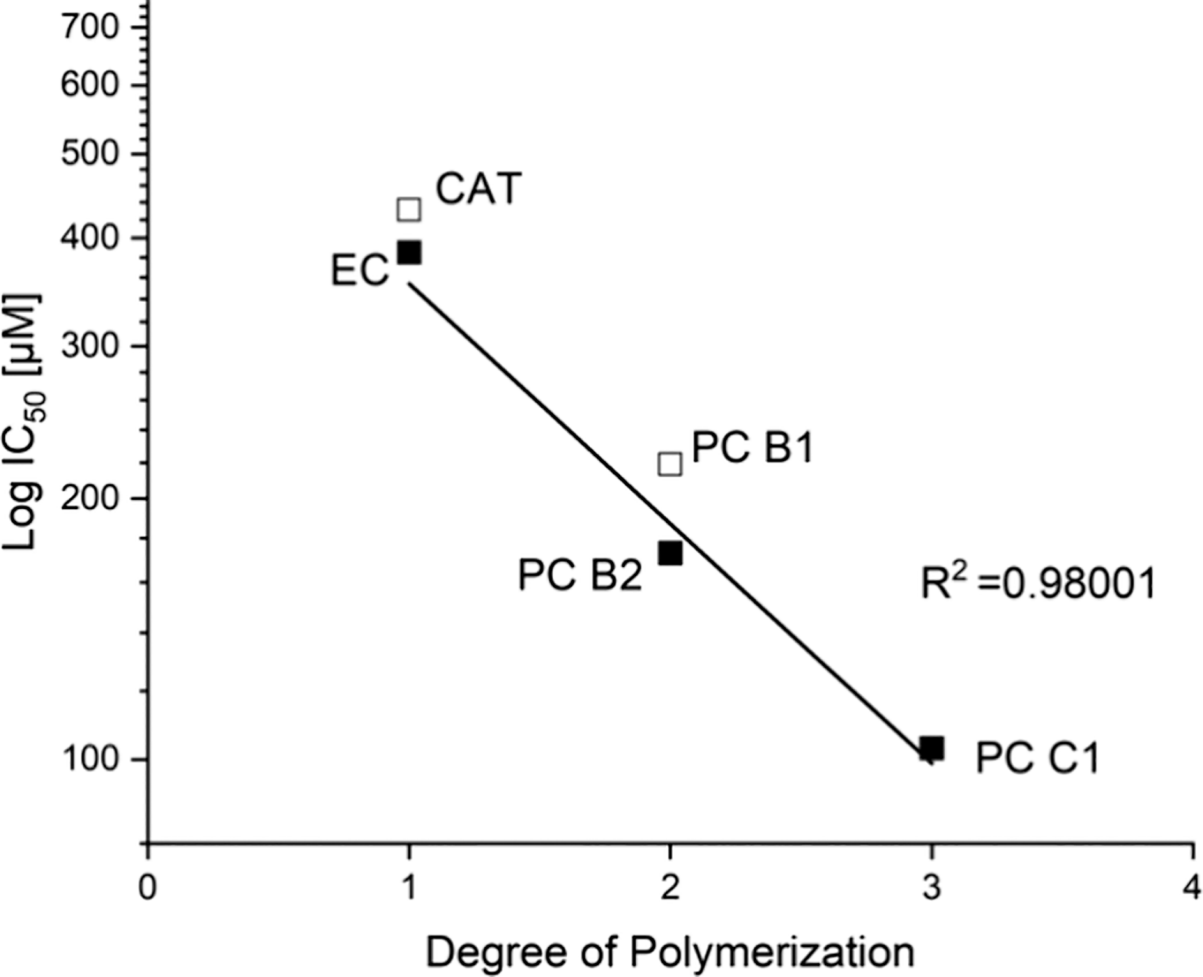
Correlation between IC_50_ values and the degree of polymerization. The coefficient of determination is calculated for the linear regression of the data related to epicatechin-based compounds.

Our findings regarding the interaction stand in contrast to the impact of the flavan-3-ols on α-amylase activity. The inhibitory strength of the flavan-3-ols exhibited a positive correlation with the number of monomer units. While the inhibition was marginal for the monomers with *K*_ic_ and *K*_iu_ values up to 650 μM, a substantial reduction in α-amylase activity by 50% was achieved with 100 μM PC C1. It is evident that CAT and PC B1 have a lower inhibitory capacity than their EC-containing counterparts (Figure 10 and S53). While a low interaction results in a low inhibition and vice versa for the monomers, no such trend is observed for the dimers.

The discrepancy between the trends observed in the interaction studies and the α-amylase inhibition requires explanation. Both ITC and STD-NMR are direct approaches to determine interactions, but they are limited to reversible binding events.^29^ Irreversible interactions and aggregate formation are not detectable by either technique. Furthermore, as indicated by the low affinity, the interaction is most likely nonspecific and only interactions close to the active site of α-amylase have a direct effect on starch hydrolysis. In contrast, the assessment of enzyme inhibition is an indirect approach that combines both reversible and irreversible interactions. With the transition from monomers to trimers, we move beyond the reversible interaction regime, where irreversible aggregation occurs. This transition is supported by the elevated loss of α-amylase activity in the presence of PC B1 and PC B2 compared to the monomers and the visible precipitation of α-amylase with PC C1 in the sample of our STD-NMR and the fluctuating heat signals in the ITC thermogram. In addition, the adsorption of PC C1 on the α-amylase surface and the formation of higher aggregates were confirmed by the decrease in proton signal intensities (Figure 6) in NMR experiments. Thus, this explains the strong impact of the DP on the inhibition of digestive enzymes, with the most effective inhibition observed for high-molecular-weight oligomers.^27^

To date, the inhibition constants can only be compared with various UV-based assays to evaluate α-amylase activity, e.g., the hydrolysis of the artificial substrate chloronitrophenylgalactopyranosylmaltoside (Gal-G_2_-CNP). In their study, Kaeswurm et al. reported *K*_ic_ and *K*_iu_ values of 88 ± 37 and 231 ± 45 μM, respectively, for inhibition of α-amylase by EC.^57^ In contrast, the current study observed a significantly reduced effect on starch conversion, with *K*_ic_ = 253 ± 47 μM and *K*_iu_ = 385 ± 82 μM. This discrepancy is likely attributed to the different substrate and the direct monitoring of its conversion by ITC in contrast to UV/Vis detection, which results in discrete values, fitted in the Michaelis–Menten diagram. Nevertheless, this discrepancy highlights the importance of using the natural substrate starch to investigate the impact of secondary plant metabolites on α-amylase inhibition. The degree of overlap between the inhibitory effects of the inhibitors and the products resulting from starch conversion appears to be less relevant due to the disparate magnitudes of inhibition.

Sun et al. studied the inhibition of α-amylase by phenolic extracts derived from young apples and compared their efficacy to that of several purified phenolic compounds.^61^ Their findings revealed that EC exhibited the weakest inhibition compared to other phenolic compounds and the complex apple extract. In their investigation of the inhibitory effect of cacao extracts on α-amylase, Gu et al. observed a negligible reduction in activity (10%) by EC, while a procyanidin decamer resulted in a 45% decrease in α-amylase activity.^27^ This aligns with the trend observed in our study, where the strength of inhibition correlates with the degree of polymerization of the procyanidins.

In conclusion, this study provides a comprehensive overview of the interaction mechanism and the resulting inhibition of α-amylase activity, which comprises competitive and uncompetitive reversible and irreversible inhibition. The data demonstrate the importance of information about the structural characteristics present under interaction conditions to gain a comprehensive understanding of the proposed beneficial effects of flavan-3-ols on the prevention of type 2 diabetes.

## Data Availability

The data generated during the study are included in this published article and its supplementary file.

## Supporting Information Available

Parameter and additional information for ITC and NMR method evaluation, NMR spectra and signal assignment data, and spectra that support the results and statements made in the main manuscript; individual thermograms and the resulting Wiseman plots for polyphenol-α-amylase interaction; S1: parameters for 2D NMR experiments acquired at 274 K and applying nonuniform sampling (NUS) for all the data; S2: optimizing α-amylase saturation in STD-NMR experiments in 0.1 M PBS/0.04 M NaCl (D_2_O/H_2_O, 90/10 v/v) at 298 K and at 700 MHz; S3: stacked STD spectra and off-resonance spectrum of 15 μM α-amylase in 0.1 M PBS/0.04 M NaCl (D_2_O/H_2_O, 90/10 v/v) at 700 MHz and at 274 K for the parameter sets 7, 8, 10, and 11 with the respective saturation percentage; S4: heat generation from dilution and mixing at 298 K; S5: stability of ligands in 0.1 M PBS at room temperature (rt) and 277 K; S6: thermogram of recurrent single injection experiments with starch (1.1 g/L per injection) titrated into 0.1 M phosphate buffer/0.04 M NaCl (blank 1) and with 0.1 M phosphate buffer/0.04 M NaCl titrated into α-amylase (12 nM) (blank 2); S7–S11: overlay of the thermogram (left) and Wiseman plot (right) of the interaction of 0.1 mM α-amylase with 4 mM EC, 4 mM CAT, 4 mM PC B1, with 4 mM PC B2, and 4 mM PC C1; S12: thermodynamic parameters of the interaction of 0.1 mM α-amylase and 4 mM flavan-3-ols; S13: proton NMR (noesygppr1d) of PC B1 in methanol-*d*_4_:D_2_O (1:1) at 700 MHz and 274 K showing shift regions of the different rings; S14: detailed signal assignment in proton spectra (noesygppr1d) of PC B1 in methanol-*d*_4_:D_2_O (1:1) at 700 MHz and 274 K. S15: overlay of ^1^H–^13^C HSQC and ^1^H–^13^C HMBC of PC B1 in methanol-*d*_4_:D_2_O (1:1) at 700 MHz and 274 K; S16 and S17: ^1^H–^1^H TOCSY spectra (mixing times, 20 and 80 ms) of PC B1 in methanol-*d*_4_:D_2_O (1:1) at 700 MHz and 274 K; S18–S21: ^1^H–^1^H ROESY spectrum, ^1^H proton and ^13^C carbon shifts, proton–proton coupling constants, and proton integrals of PC B1 in methanol-*d*_4_:D_2_O (1:1) at 700 MHz and 274 K; S22–S24: ^1^H–^1^H TOCSY spectrum (mixing time, 100 ms), proton shifts and integrals, and proton–proton coupling constants of PC B1 in 0.1 M PBS/D_2_O at 700 MHz and 274 K; S25: proton NMR (noesygppr1d) of PC B2 in methanol-*d*_4_:D_2_O (1:1) at 700 MHz and 274 K showing shift regions of the different rings; S26–S31: detailed signal assignment in proton spectra (noesygppr1d), overlay of ^1^H–^13^C HSQC and ^1^H–^13^C HMBC, ^1^H–^1^H TOCSY spectrum (mixing time, 20 ms), ^1^H–^1^H NOESY spectrum, ^1^H proton and ^13^C carbon shifts, and ^1^H–^1^H coupling constants of PC B2 in methanol-*d*_4_:D_2_O (1:1) at 700 MHz and 274 K; S32: integrals of PC B2 and the ratio between the rotamers in methanol-*d*_4_:D_2_O (1:1) at 700 MHz and 274 K; S33–S36: ^1^H–^1^H TOCSY spectrum (mixing time, 100 ms), proton shifts, proton–proton coupling constants, and integrals of PC B2 in 0.1 M PBS/D_2_O at 700 MHz and 274 K; S37: proton NMR (noesygppr1d) of PC C1 in methanol-*d*_4_:D_2_O (1:1) at 700 MHz and 274 K showing shift regions of the different rings; S38–S40: detailed signal assignment in proton spectra (noesygppr1d), proton shifts and integrals and ^13^C carbon shifts, and proton–proton coupling constants of PC C1 in methanol-*d*_4_:D_2_O (1:1) at 700 MHz and 274 K; S41 and S42: ^1^H–^1^H TOCSY spectrum (mixing time, 100 ms) and proton shifts and integrals of PC C1 in 0.1 M PBS/D_2_O at 700 MHz and 274 K; S43: stacked STD spectra of 10 μM α-amylase with 1.3 mM CAT in 0.1 M PBS/0.04 M NaCl (D_2_O/H_2_O, 90/10 v/v) and off-resonance spectrum of 1.3 mM CAT at 700 MHz and at 298 K; S44: stacked STD spectra of 10 μM α-amylase with 0.9 mM PC B1 in 0.1 M PBS/0.04 M NaCl (D_2_O/H_2_O, 90/10 v/v) and off-resonance spectrum of 0.9 mM PC B1 at 700 MHz and at 298 K; S45: stacked STD spectra of 10 μM α-amylase with 0.9 mM PC B2 in 0.1 M PBS/0.04 M NaCl (D_2_O/H_2_O, 90/10 v/v) and off-resonance spectrum of 0.9 mM PC B2 at 700 MHz and at 298 K; S46: stacked STD spectra of 10 μM α-amylase with 1.0 mM PC C1 in 0.1 M PBS/0.04 M NaCl (D_2_O/H_2_O, 90/10 v/v) and off-resonance spectrum of 1.0 mM PC C1 at 700 MHz and at 298 K; S47: full proton spectra (zgesgp) of PC C1 and changes in intensities by addition of 20 μM α-amylase at 275 K in 100 mM phosphate buffer (D_2_O:H_2_O 9:1, 40 mM NaCl, pH 7); S48: overlay of PC C1 and α-amylase proton spectra (zgesgp) and mixtures thereof at 275 K in 100 mM phosphate buffer (D_2_O:H_2_O 9:1, 40 mM NaCl, pH 7); S49: calculated secondary protein structures of the different samples; S50: thermogram of recurrent single injection experiments with starch (1.1 g/L (5 mM) per injection) titrated into α-amylase (12 nM) at 310 K and overlay of injections (control); S51: changes in inhibition parameters calculated for the flavan-3-ols (100 μM) with an increasing impact of product inhibition from injection (inj.) 1 to 3; S52: thermogram of starch conversion (1.1 g/L (5 mM), 310 K) after incubation of 12 nM α-amylase with 0, 100, and 105 μM PC C1; S53: correlation of IC_50_ values with the molecular weight (PDF)

## Abbreviations

HMBC: heteronuclear multiple bond correlation
HSQC: heteronuclear single quantum correlation
NOESY: nuclear Overhauser enhancement spectroscopy
ROESY: rotating-frame Overhauser enhancement spectroscopy
TOCSY: total correlation spectroscopy
*c*: Wiseman parameter
CAT: (+)-catechin
SD: standard deviation
DFT: density functional theory
DOP: degree of polymerization
EC: (−)-epicatechin
EI: enzyme–inhibitor complex
ES: enzyme–substrate complex
ESI: enzyme–substrate–inhibitor complex
*I*: inhibitor concentration
inj.: injection
ITC: isothermal titration calorimetry
*K*_a_: association constant
*k*_cat_: catalytic rate constant
*K*_d_: dissociation constant
*K*_ic_: competitive inhibition constant
*K*_iu_: uncompetitive inhibition constant
*K*_m_: Michaelis–Menten constant
*v*_max_: maximal reaction rate
MM: Michaelis–Menten
*K*_m_: Michaelis–Menten constant
MW: molecular weight
*n*: stoichiometry
NOE: nuclear Overhauser effect
PC B1: procyanidin B1
PC B2: procyanidin B2
PC C1: procyanidin C1
*Q*: heat
S: substrate
STD-NMR: saturation transfer difference-nuclear magnetic resonance
*V*_cell_: volume of the sample cell
Δ*G*: Gibbs free energy
Δ*H*: enthalpy
Δ*H*_app_: experimentally determined molar enthalpy
Δ*S*: entropy
