# Supplementary material for: Applying Isothermal Titration Calorimetry and Saturation Transfer Difference-NMR to Study the Mode of Interaction of Flavan-3-ols with α-Amylase to Understand Their Impact on Starch Hydrolysis
Source: J Agric Food Chem. 2025 Apr 4;73(15):9047–61. doi: 10.1021/acs.jafc.4c13178 (PMC12007089; doi:10.1021/acs.jafc.4c13178)

Supplemental Material

Applying Isothermal Titration Calorimetry and Saturation Transfer Difference-NMR to Study the Mode of Interaction of Flavan-3-ols with *α*-Amylase to Understand Their Impact on Starch Hydrolysis

Birgit Claasen[1], Mengyao Xiong[2,3], Pia S. Mayer[2], Greta Sogl[2], and Maria Buchweitz[2,3]*

[1] Analytical Department, Institute of Organic Chemistry, University of Stuttgart, Stuttgart 70569, Germany.

[2] Department of Food Chemistry, Institute of Biochemistry and Technical Biochemistry, University of Stuttgart, Stuttgart 70569, Germany.

[3] Institute of Food Chemistry, University of Hamburg, Hamburg 20146, Germany.

*Corresponding author: M. Buchweitz

Phone: +49 40 42838 7979; Email: maria.buchweitz@uni-hamburg.de

---

S1: Parameters for 2D NMR experiments acquired at 274K and applying nonuniform sampling (NUS) for all the data.

solvent	ligand	spectrum	pulse program	mixing time [ms]	acquisition (TD)		processing (ST)	
					F2	F1	F2	F1
		^1H-^1H				256		512
**0,1 M PBS/D_2O**	PC B1	TOCSY	mlevphpr.2	100	4096		4096	
		^1H-^1H			4096	256	4096	512
	PC B2	TOCSY	mlevphpr.2	100				
		^1H-^1H			4096	512	4096	512
	PC C1	TOCSY	mlevphpr.2	100				
**Methanol-**	PC B1	^1H-^{13}C	Hmbcgplpndprqf	-	4096	512	8192	512
**d_4:D_2O**		HMBC						
**(1:1)**		^1H-^{13}C	hsqcedetgpsisp2.		4096	128	4096	512
		HSQC	3	-				
		^1H-^1H			4096	256	4096	512
		TOCSY	Mlevphpp	20				
		^1H-^1H			4096	256	4096	512
		TOCSY	Mlevphpp	80				
		^1H-^1H			4096	256	4096	512
		ROESY	roesyphpr.2	800				
		^1H-^{13}C			4096	512	4096	512
	PC B2	HMBC	Hmbcgplpndprqf	-				
		^1H-^{13}C	hsqcedetgpsisp2.		4096	512	4096	512
		HSQC	3	-				
		^1H-^1H			4096	256	4096	512
		TOCSY	mlevgpphw5	20				
		^1H-^1H			4096	256	4096	512
		NOESY	Noesyphpr	-				
		^1H-^{13}C			8192	512	4096	1024
	PC C1	HMBC	Hmbcgplpndprqf	-				
		^1H-^{13}C	hsqcedetgpsisp2.		4096	512	4096	1024
		HSQC	3	-				
		^1H-^1H			4096	512	8192	1024
		TOCSY	mlevphpr.2	30				
		^1H-^1H			8192	512	8192	1024
		TOCSY	mlevphpr.2	100				
		^1H-^1H			4096	512	4096	1024
		ROESY	roesyphpr.2	300				

S2: Optimizing α-amylase saturation in STD-NMR experiment in 0.1 M PBS/ 0.04M NaCl (D_2O/H_2O, 90/10 v/v) at 298 K and at 700 MHz. A series of varied parameters was tested to assess their impact on saturation. The optimal set of parameters, as indicated, was chosen for protein-ligand measurements.

parameter set	saturation [%]	FQ2 [ppm]	D20 [s]	SPW 9 [dB]	P42 [ms]
1	**15**	0	2	45	50
2	**42**	0.5	2	45	50
3	**35**	0	2	40	50
4	**50**	0	2	35	50
5	**21**	0	3	45	50
6	**25**	0	2	45	40
7	**25**	0	3	35	50
8	**60**	0.5	3	35	50
9	**11**	-0.5	2	45	50
10	**70**	1	3	35	50
11	**75**	1.5	3	35	50

S3: Stacked STD spectra and an off-resonance spectrum of 15 µM α-amylase in 0.1 M PBS/ 0.04M NaCl (D_2O/H_2O, 90/10 v/v) at 700 MHz and at 274 K for the parameter sets 7, 8, 10 and 11 with the respective saturation percentage.

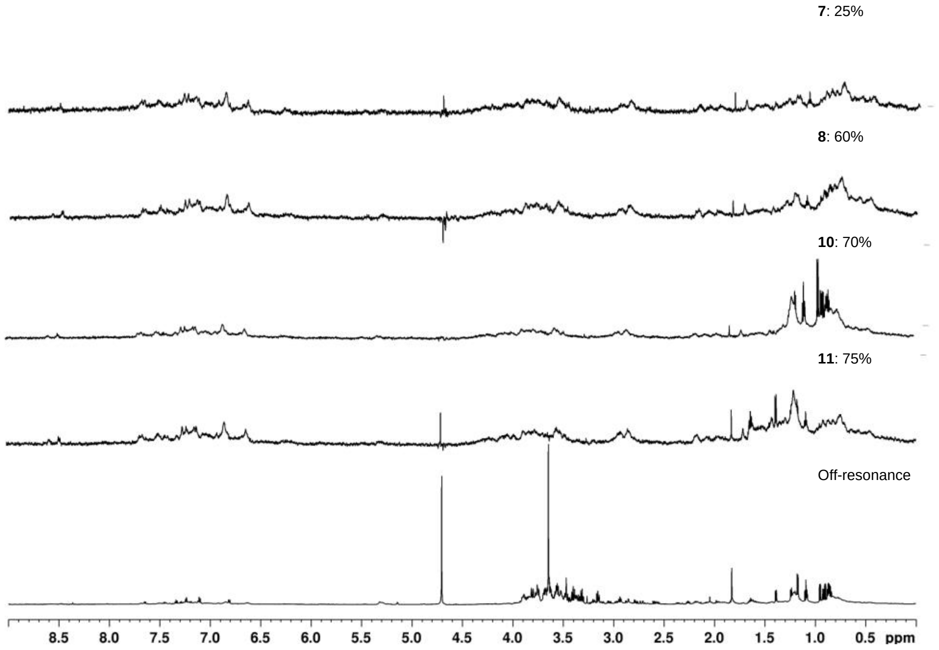

S4: Heat generation from dilution and mixing at 298 K.

**syringe into cell** (4 mM flavan-3-ols)	**max. heat rate**[1] [µJ/s]	**heat area**[2] [µJ]
buffer into amylase	-0,27±0,10	6,52±0,98
buffer into buffer	0,07±0,01	0,86±0,09
EC into buffer	0,10±0,01	2,03±0,14
CAT into buffer	0,56±0,03	8,08±0,89
PC B1 into buffer	0,19±0,01	3,10±0,29
PC B2 into buffer	0,11±0,01	2,19±0,53

[1]intensity of the peak; [2]heat development; mean ± average deviation, n= 2.

S5: Stability of ligands in 0.1 M PBS at room temperature (rt) and 277 K.

**polyphenol**	**temp**	**conc.**[1] [mM]	**rel. dev.** [%]	**storage time** [h]	**difference rt and 277 K** [mM]
**EC**[2]	rt	4,23 ± 0,24	6	3,5	
	277 K	3,99 ± 0,25	6	3,5	0,24
**CAT**[3]	rt	4,02 ± 0,21	5	22	
	277 K	3,94 ± 0,24	6	22	0,08
**PC B1**[3]	rt	4,10 ± 0,29	7	15	
	277 K	4,28 ± 0,20	5	15	0,18
**PC B2**[3]	rt	4,56 ± 0,21	5	15	
	277 K	4,46 ± 0,24	5	15	0,11
**PC C1**[3]	rt	4,29 ± 0,30	7	15	
	277 K	4,60 ± 0,42	9	15	0,31

[1]average ± SD, [2] n = 4, [3] n = 6

S6: Thermogram of recurrent single injection experiment with starch (1.1 g/L per injection) titrated into 0.1 M phosphate buffer/ 0.04 M NaCl (blank 1) and with 0.1 M phosphate buffer/ 0.04 M NaCl titrated into α-amylase (12 nM) (blank 2).

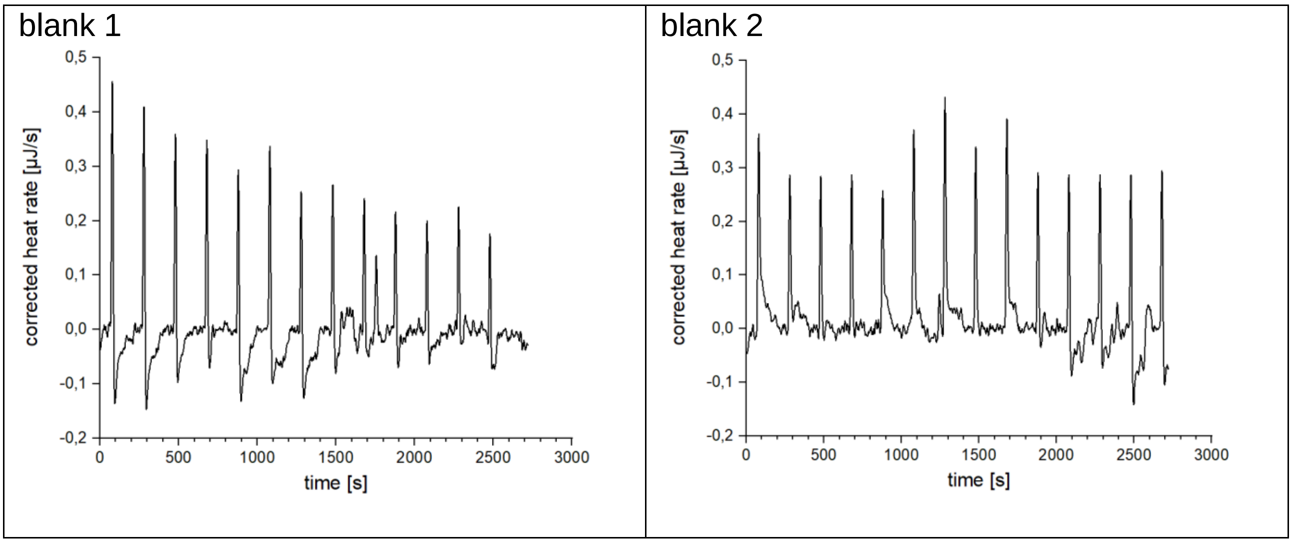

Interaction studies of flavan-3-ols with α-amylase by ITC

S7: Overlay of the thermogram (left) and Wiseman plot (right) of the interaction of 0.1 mM α-amylase with 4 mM EC.

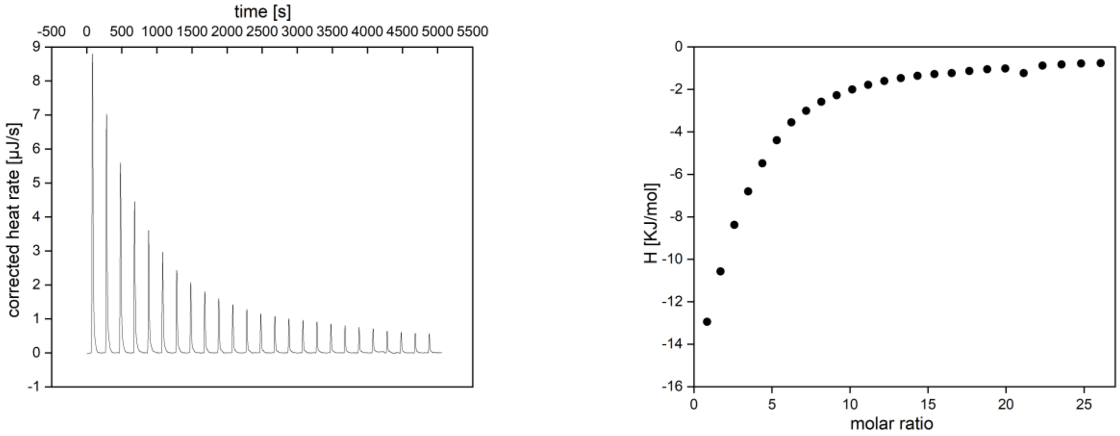

S8: Overlay of the thermogram (left) and Wiseman plot (right) of the interaction of 0.1 mM α-amylase with 4 mM CAT.

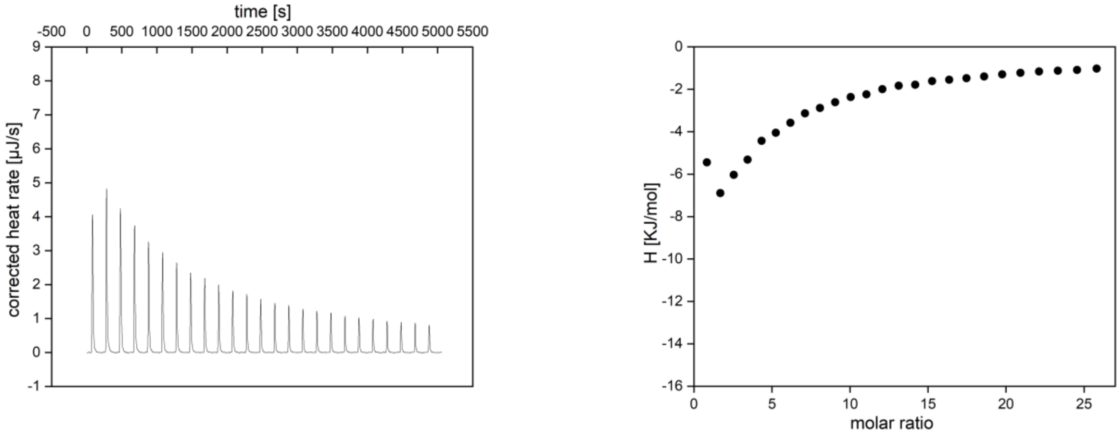

S9: Overlay of the thermogram (left) and Wiseman plot (right) of the interaction of 0.1 mM α-amylase with 4 mM PC B1.

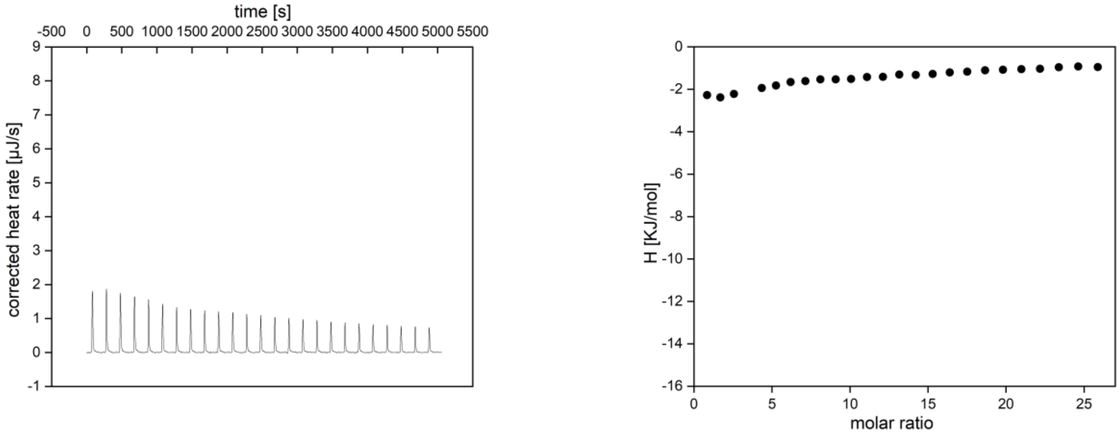

S10: Overlay of the thermogram (left) and Wiseman plot (right) of the interaction of 0.1 mM α-amylase with 4 mM PC B2.

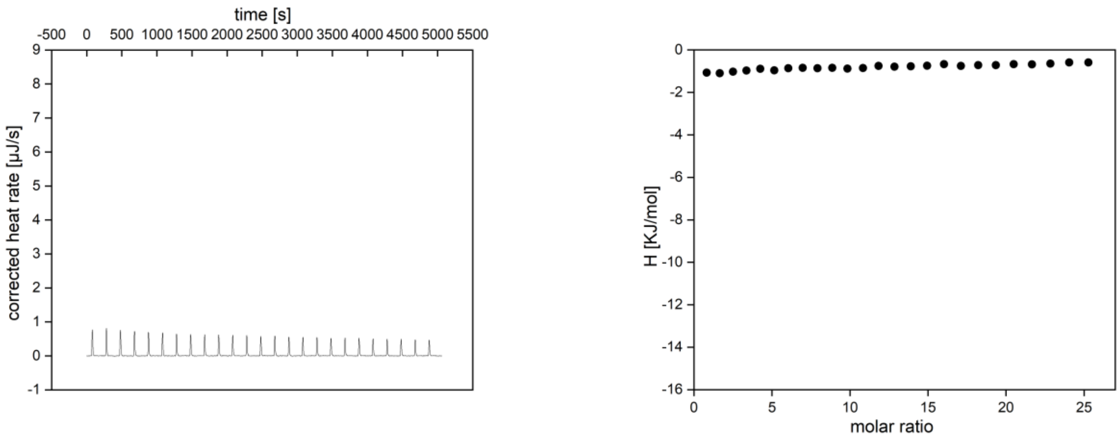

S11: Overlay of the thermogram (left) and Wiseman plot (right) of the interaction of 0.1 mM α-amylase with 4 mM PC C1.

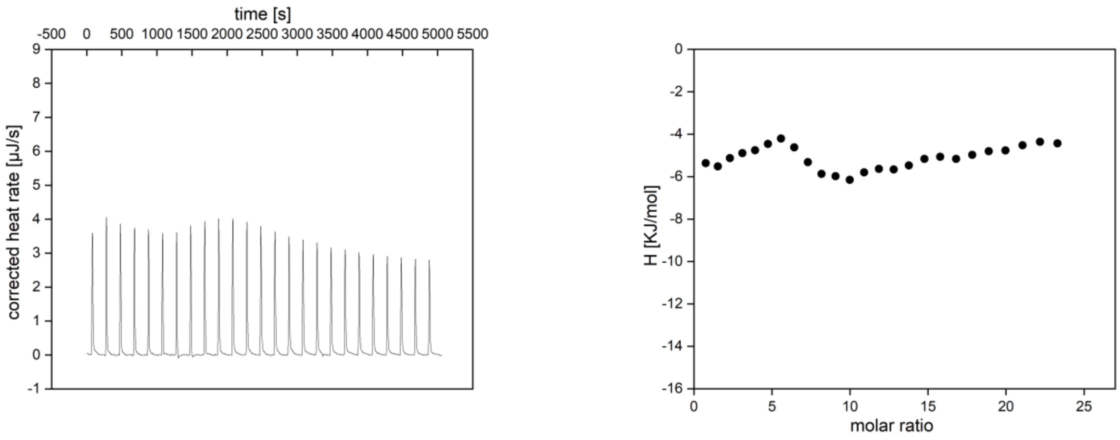

S12: Thermodynamic parameters of the interaction of 0.1 mM α-amylase and 4 mM flavan-3-ols.

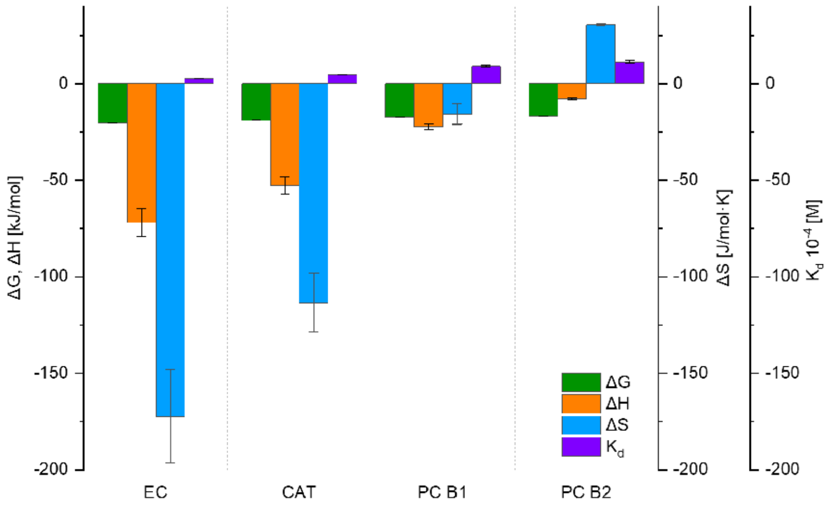

Signal assignment PC B1

S13: Proton NMR (noesygppr1d) of PC B1 in methanol-d$_4$:D$_2$O (1:1) at 700 MHz and 274 K showing shift regions of the different rings.

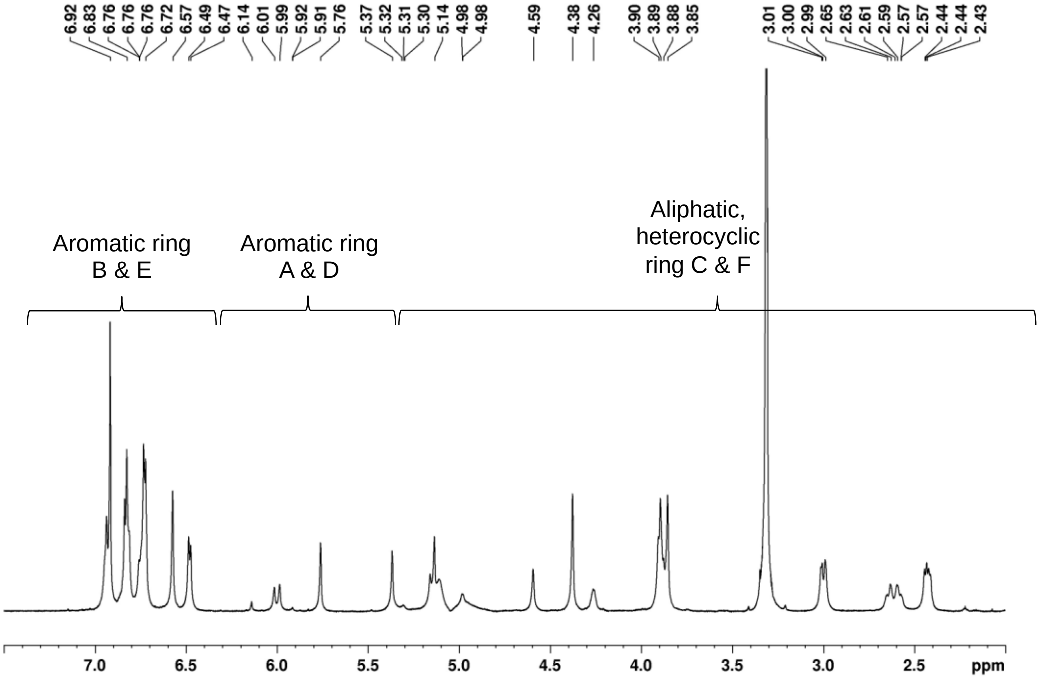

S14: Detailed signal assignment in proton spectra (noesygppr1d) of PC B1 in methanol-d$_4$:D$_2$O (1:1) at 700 MHz and 274 K.

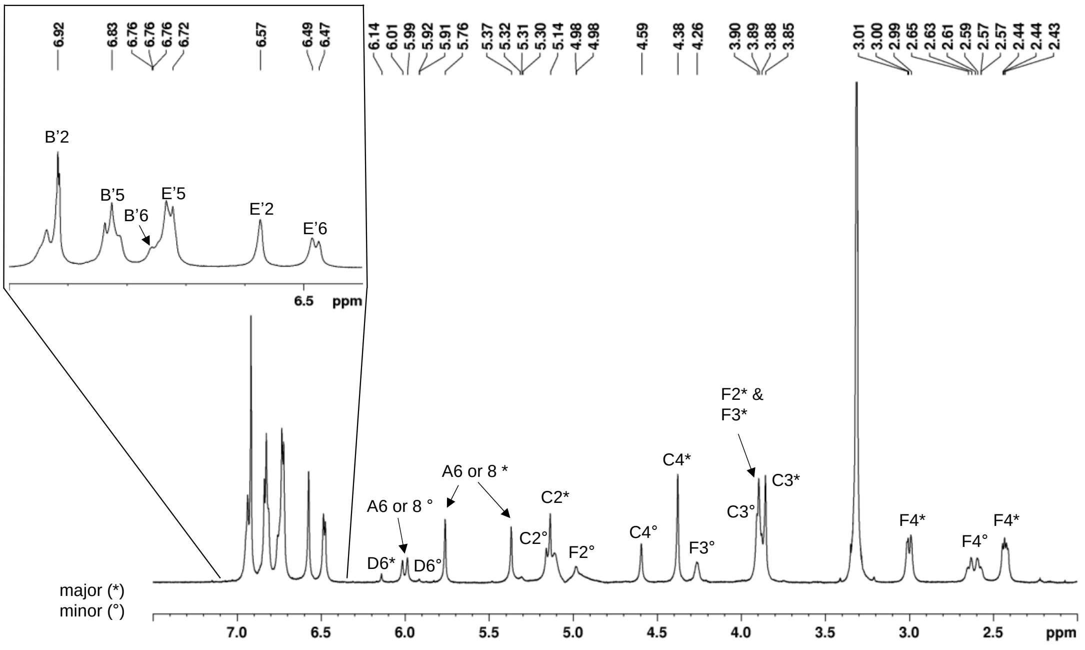

S15: Overlay of ^{1}H – ^{13}C HSQC (pulse sensitive, positive: light green; negative: dark green) and ^{1}H – ^{13}C HMBC (black) of PC B1 in methanol-d_4:D_2O (1:1) at 700 MHz and 274 K.

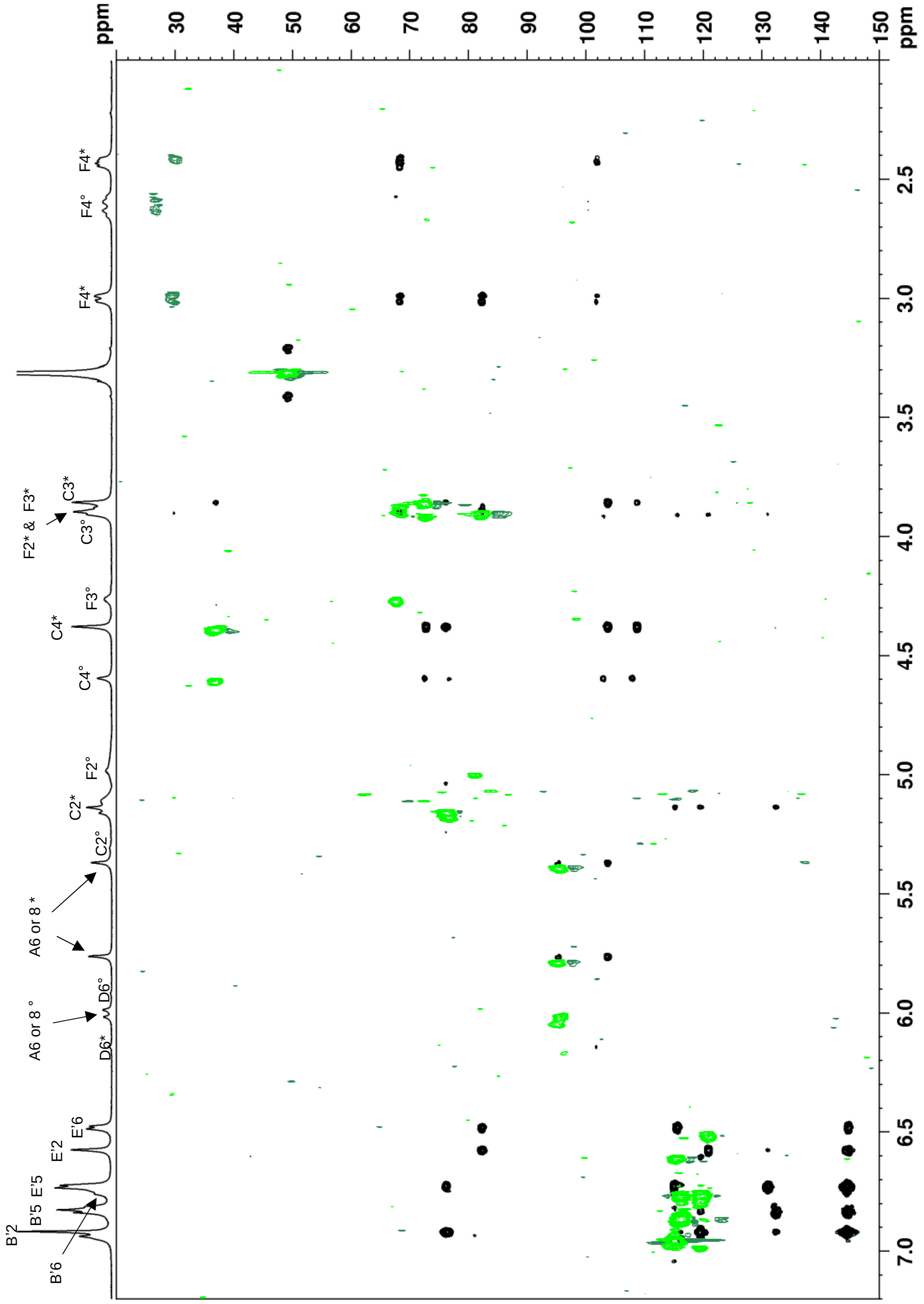

S16: ^{1}H – ^{1}H TOCSY spectrum (mixing time 20 ms, positive contour: black, negative contour: red) of PC B1 in methanol-d_4:D_2O (1:1) at 700 MHz and 274 K.

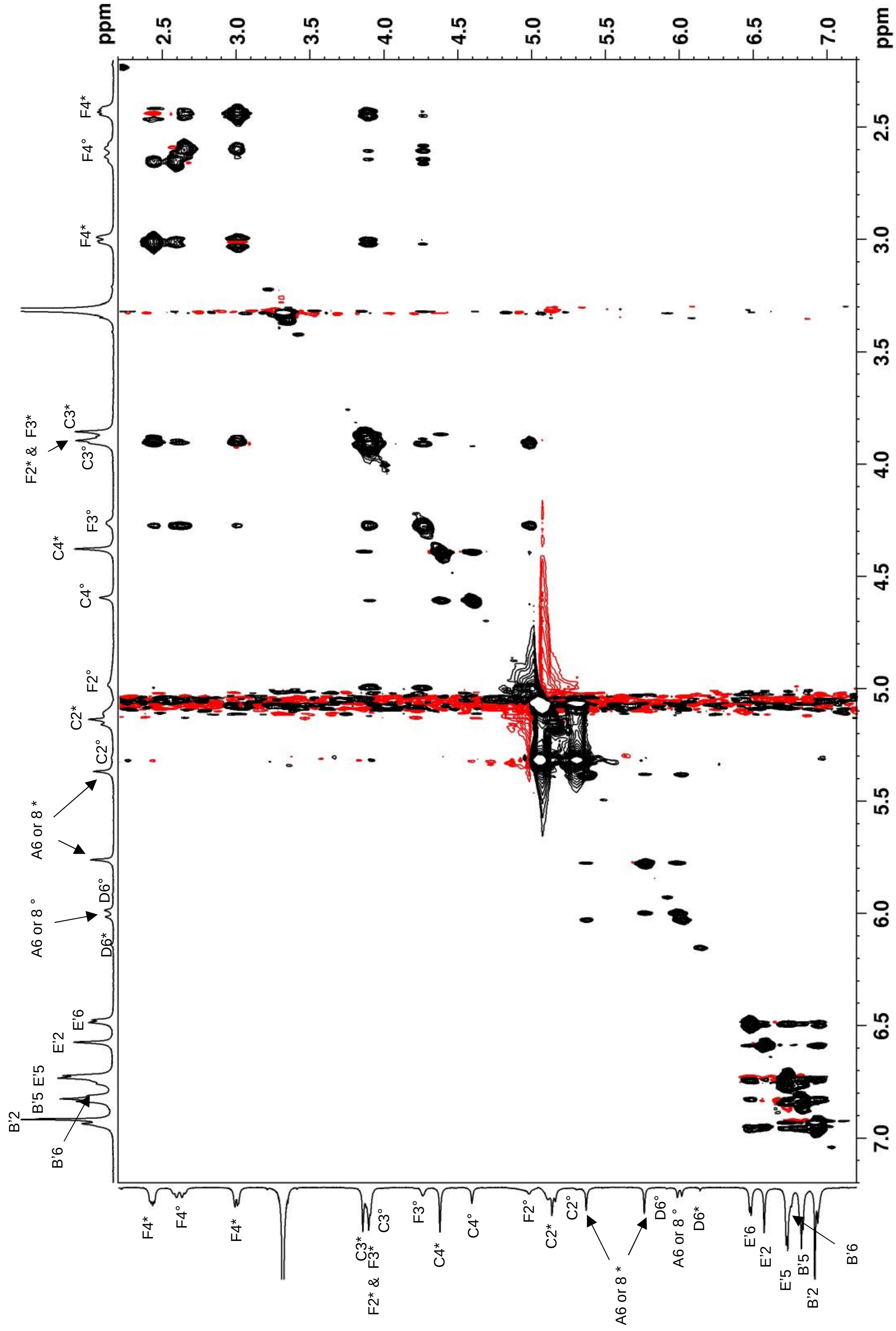

S17: ^1H – ^1H TOCSY spectrum (mixing time 80 ms, positive contour: black, negative contour: red) of PC B1 in methanol-d_4:D_2O (1:1) at 700 MHz and 274 K.

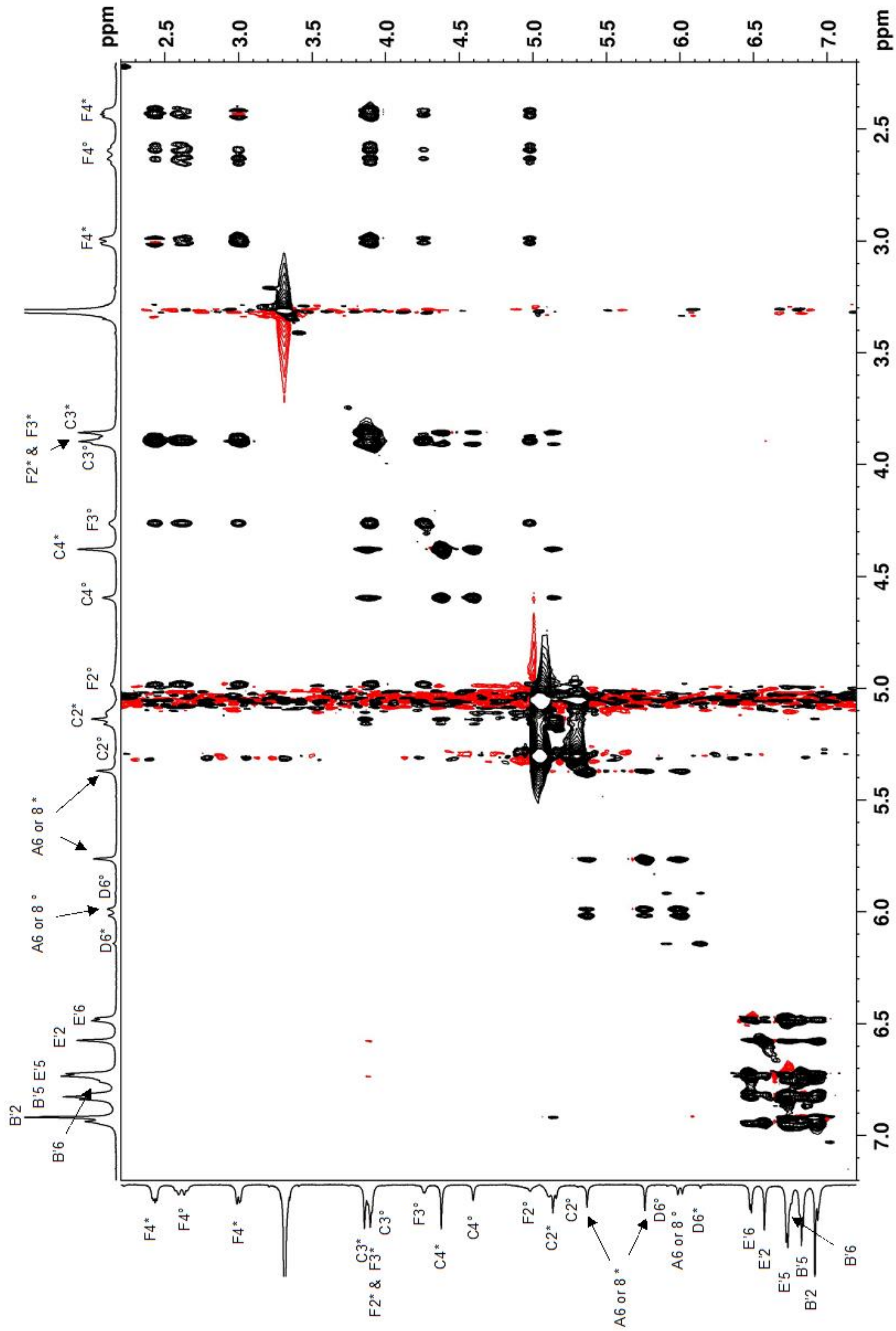

S18: $^{1}H - ^{1}H$ ROESY spectrum (mixing time 800 ms, positive contour: blue, negative contour: purple) of PC B1 in methanol-d_4:D_2O (1:1) at 700 MHz and 274 K.

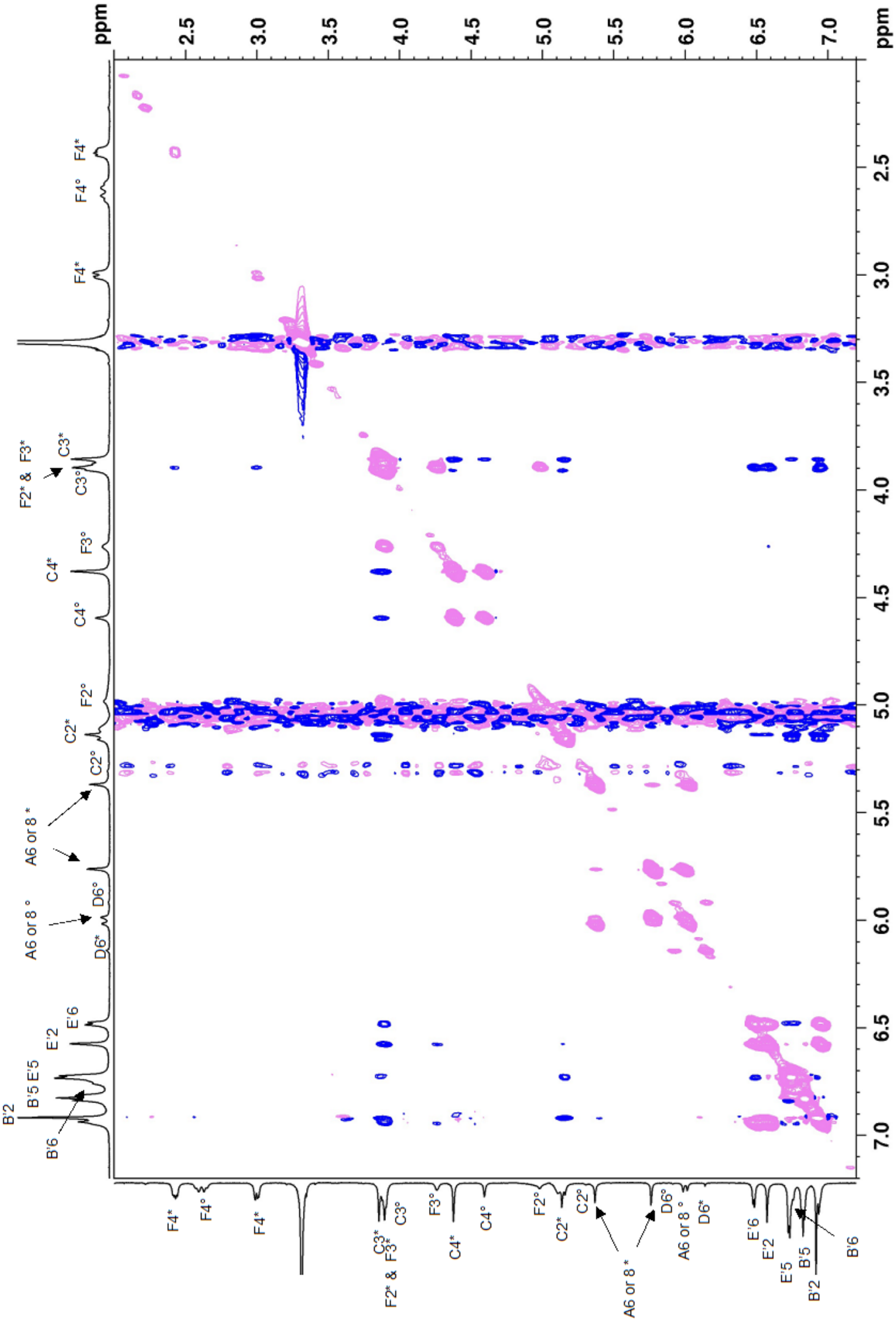

S19: ^{1}H proton and ^{13}C carbon shifts for PC B1 in methanol-d_4:D_2O (1:1) at 700 MHz and 274 K.

		^{1}H [ppm]				^{13}C [ppm]	
		**major (*)**		**minor (°)**		**major (*)**	**minor (°)**
**A**	5					153.54 - 156.9[6]	n.i.[7]
	6	5.36/5.76	s[1]	5.99/6.01	s[1]	95.54/95.63[1]	95.44/96.11[1]
	7					153.54 - 156.9[6]	n.i.[7]
	8	5.36/5.76	s[1]	5.99/6.01	s[1]	95.54/95.63[1]	95.44/96.11[1]
	9					104	103.1
	10					153.54 - 156.9[6]	n.i.[7]
**C**	2	5.13	s[2,3]	5.13	s[2,3]	76.57	76.57
	3	3.85	m[4]	3.90	m[4]	72.76[3]	72.76[3]
	4	4.38	s	4.59	s	37.05	36.83
**B**	1´					133.11	n.i.[7]
	2´	6.92				115.49	n.i.[7]
	3´					145.46	n.i.[7]
	4´					145.08	n.i.[7]
	5´	6.83	m			116.53	n.i.[7]
	6´	6.76	m[5]			120.06	n.i.[7]
**D**	5					153.54 - 156.9[6]	n.i.[7]
	6	6.14	s	5.76	s	96.16	n.i.[7]
	7					153.54 - 156.9[6]	n.i.[7]
	8					108.9	108.07
	9					102.05	n.i.[7]
	10					153.54 - 156.9[6]	n.i.[7]
**F**	2	3.89	m[4]	4.98	s[2]	82.78[3]	81.34
	3	3.89	m[4]	4.26	s	68.33[3]	67.78
	4	2.43 & 3.00	2x dd	2.58 & 2.63	2x d	29.65 & 30.11	26.66
**E**	1´					131.53	n.i.[7]
	2´	6.57	s			115.98	n.i.[7]
	3´					145.4	n.i.[7]
	4´					145.35	n.i.[7]
	5´	6.74	m[5]			116.42	n.i.[7]
	6´	6.48	d			121.27	n.i.[7]

[1] A6 or A8; [2] water supressed region; [3] overlap of C2*/°; [3] overlap F2*, F3*, C3* and C3°; [5] overlap B6’ and E5’; [6] identification is impossible; [7] signals of the minor rotamer are too small to be identified unambiguously

S20: Proton – proton coupling constants for PC B1 in methanol-d_4:D_2O (1:1) at 700 MHz and 274 K.

	proton - proton coupling J [Hz]	
	major (*)	minor (°)
$^3J_{B5' - B6'}$	n.d.	n.d.
$^3J_{B6' - B5'}$	n.d.	n.d.
$^3J_{C2 - C3}$	n.d.	n.d.
$^3J_{C3 - C4}$	n.d.	n.d.
$^3J_{E5' - E6'}$	n.d.	n.d.
$^3J_{E6' - E5'}$	7.8	n.d.
$^3J_{F2 - F3}$	n.d.	n.d.
$^3J_{F3 - F4}$	n.d.	n.d.
$^2J_{F4_a - F4_b}$	16.0	13.1

n.d. not detectable

S21: Proton integrals for PC B1 in methanol-d_4:D_2O (1:1) at 700 MHz and 274 K.

		integrals [rel.]			
		major (*)		minor (°)	
A	5				
A	6	0.44	s[1]	0.20 / 0.20	[1]
	7				
	8	0.44	s[1]	0.20 / 0.20	[1]
	9				
	10				
C	2	n.d.		n.d.	
	3	3.44	i.o.[2] (4H)	3.44	i.o[2] (4H)
	4	0.85		0.31	
B	1´				
B	2´	1.28	i.o. (1H)	n.d.	
	3´				
	4´				
	5´	1.86	i.o. (1H)	n.d.	
	6´	2.57	i.o.[3] (2H)	n.d.	
D	5				
D	6	0.02		0.01	
	7				
	8				
	9				
	10				
F	2	3.44	i.o.[2] (4H)	n.d.	
	3	3.44	i.o.[2] (4H)	0.45	
	4	1.10 & 1.04		0.98	**ratio 67:33**
E	1´				
	2´	0.92		n.d.	
	3´				
	4´				
	5´	2.57	i.o.[3] (2H)	n.d.	
	6´	0.96		n.d.	

[1] A6 or A8, i.o. integral overlap, superscript provides number of overlapping integrals, numbers of protons are given in brackets.

S22: ^{1}H – ^{1}H TOCSY spectrum (mixing time 100 ms, positive contour: black, negative contour: red) of PC B1 in 0.1 M PBS/D_2O at 700 MHz and 274 K.

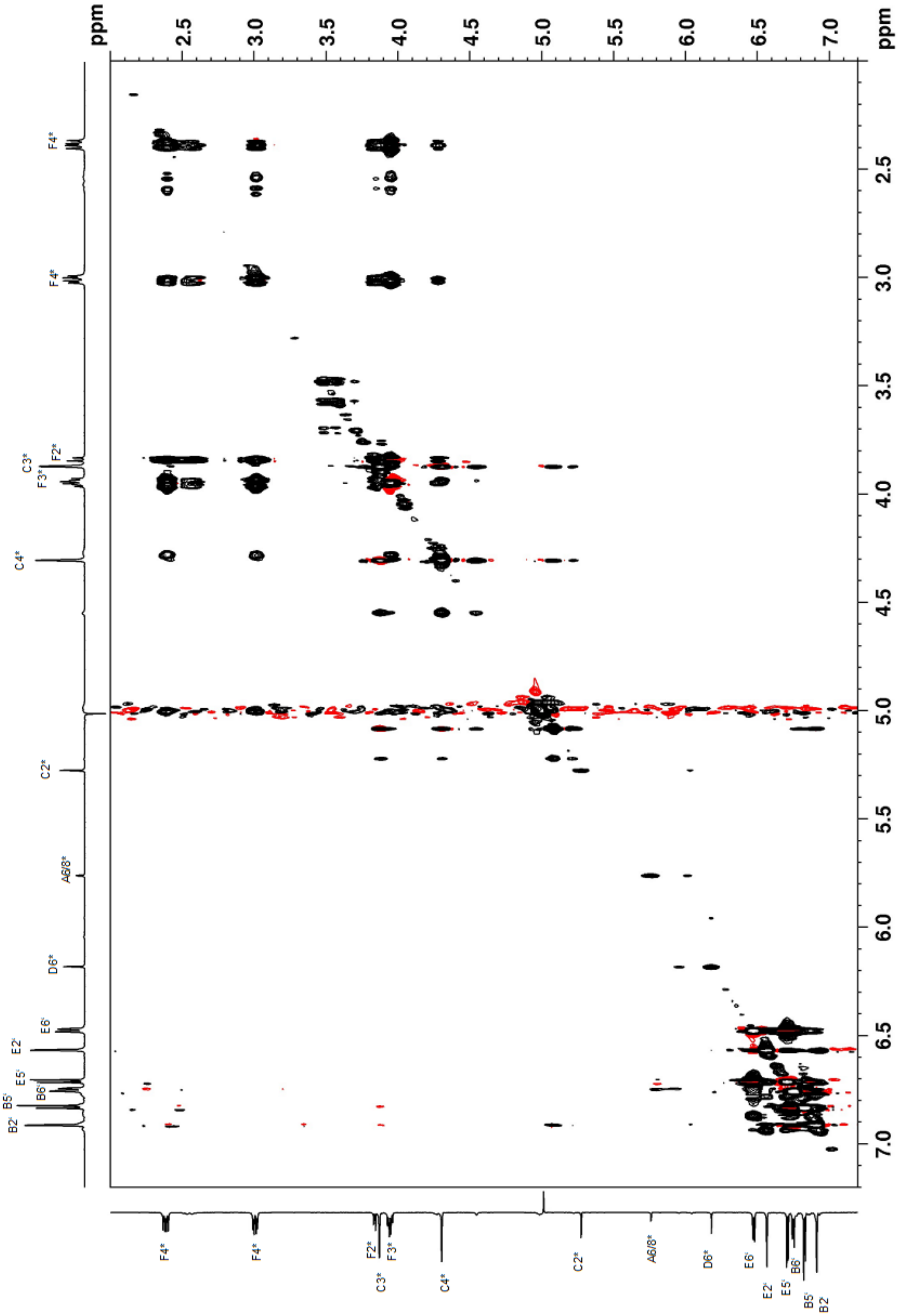

S23: Proton shifts and integrals for PC B1 in 0.1 M PBS/D_2O at 700 MHz and 274 K.

		^1H [ppm]		integrals [rel.]	
A	5				
A	6	5.76	s[1]	0.10	
	7				
	8	5.76	s[1]	0.10	
	9				
	10				
C	2	5.27	s	n.d.	
	3	3.86	s	0.81	
	4	4.3	s	0.78	
B	1´				
B	2´	6.91	s	0.92	
	3´				
	4´				
	5´	6.83	dd	0.90	
	6´	6.75	d	1.00	
D	5				
D	6	6.18	s	0.18	
	7				
	8				
	9				
	10				
F	2	3.83	d	0.13	
	3	3.95	td	1.00	
	4	2.38; 3.00	2x dd	1.00, 1.02	**ratio 100:0**
E	1´				
E	2´	6.56	s	0.80	
	3´				
	4´				
	5´	6.71	d	0.82	
	6´	6.48	d	0.83	

[1] A6/ A8 protons are not unambiguously identified and only one signal is perceptible.

S24: Proton – proton coupling constants for PC B1 in 0.1 M PBS/D_2O at 700 MHz and 274 K.

proton - proton coupling J [Hz]	
^4J B2' - B6'	1.8
^3J B5' - B6'	8.3
^3J B6' - B5'	8.3
^4J B6‘ - B2'	1.8
^3J C2 - C3	n.d.
^3J C3 - C4	n.d.
^4J E2' - E6'	1.7
^3J E5' - E6'	8.1
^3J E6' - E5'	8.2
^4J E6' - E2'	1.8
^3J F2 - F3	9.5
^3J F3 - F2	9.4
^3J F3 - $F4_a$	9.6
^3J F3 - $F4_b$	6.6
^2J $F4_a$ - $F4_b$	16.1
^3J $F4_a$ - F3	9.7
^3J $F4_b$ - F3	6.2

n.d. not detectable

Signal assignment PC B2

S25: Proton NMR (noesygppr1d) of PC B2 in methanol-d_4:D_2O (1:1) at 700 MHz and 274 K showing shift regions of the different rings.

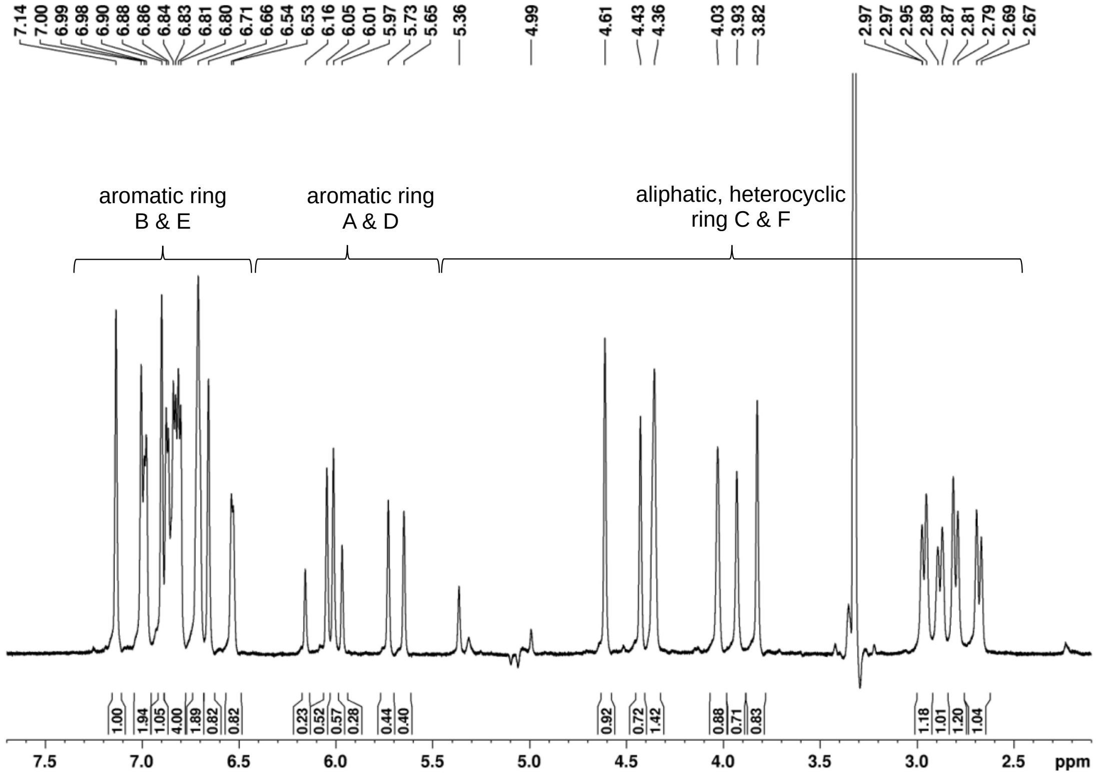

S26: Detailed signal assignment in proton spectra (noesygppr1d) of PC B2 in methanol-d_4:D_2O (1:1) at 700 MHz and 274 K.

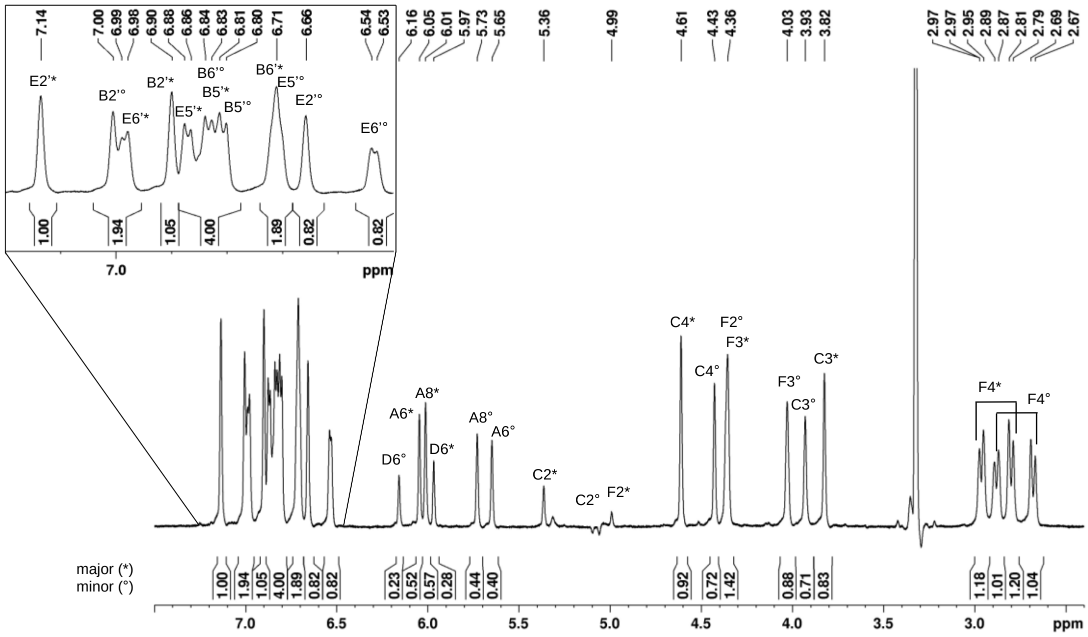

S27: Overlay of ^{1}H – ^{13}C HSQC (positive: light green; negative: dark green) and ^{1}H – ^{13}C HMBC (black) of PC B2 in methanol-d_4:D_2O (1:1) at 700 MHz and 274 K.

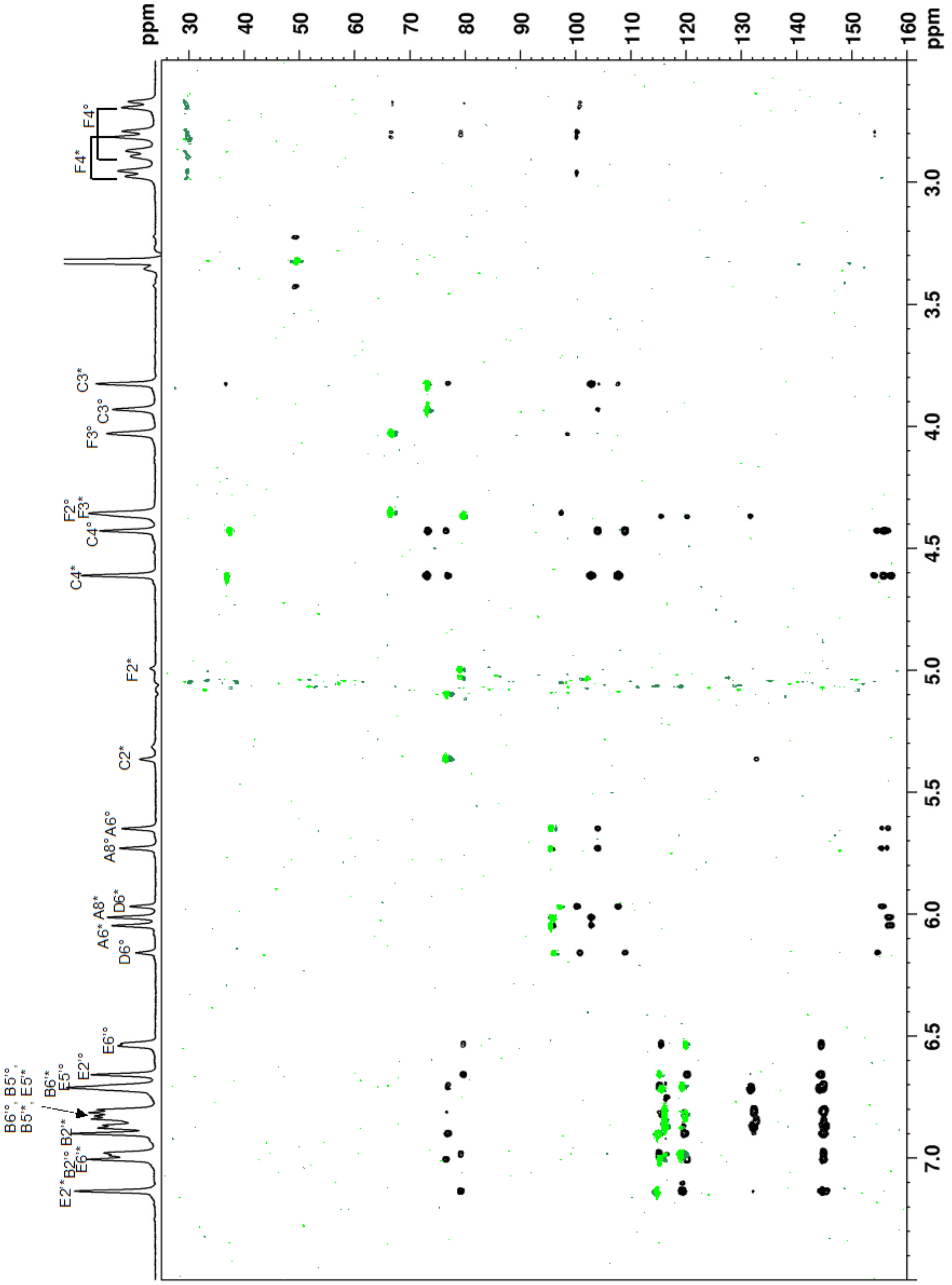

S28: ^1H – ^1H TOCSY spectrum (mixing time 20 ms, positive contour: black, negative contour: red) of PC B2 in methanol-d_4:D_2O (1:1) at 700 MHz and 274 K.

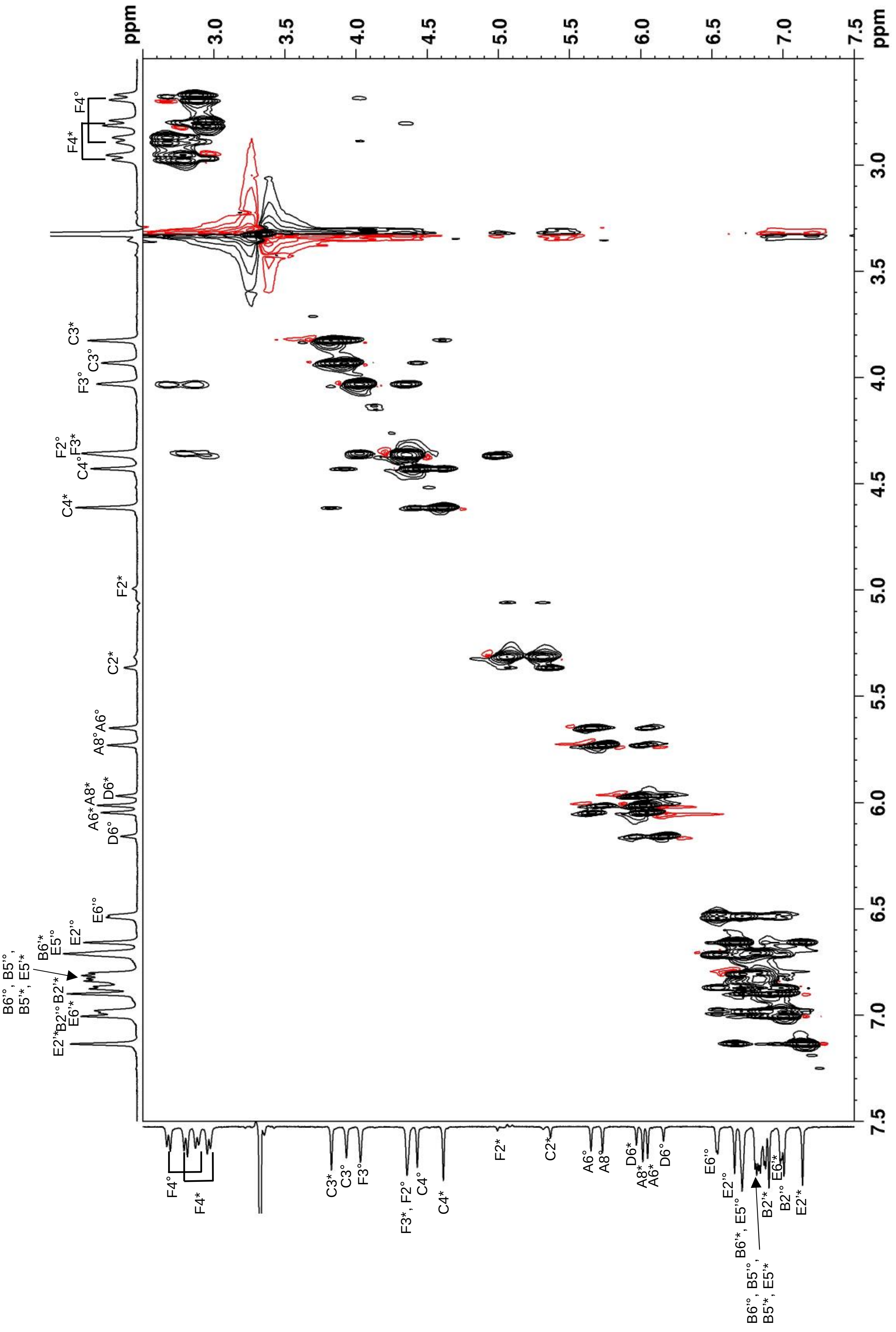

S29: ^{1}H – ^{1}H NOESY spectrum of PC B2 in methanol-d_4:D_2O (1:1) at 700 MHz and 274 K.

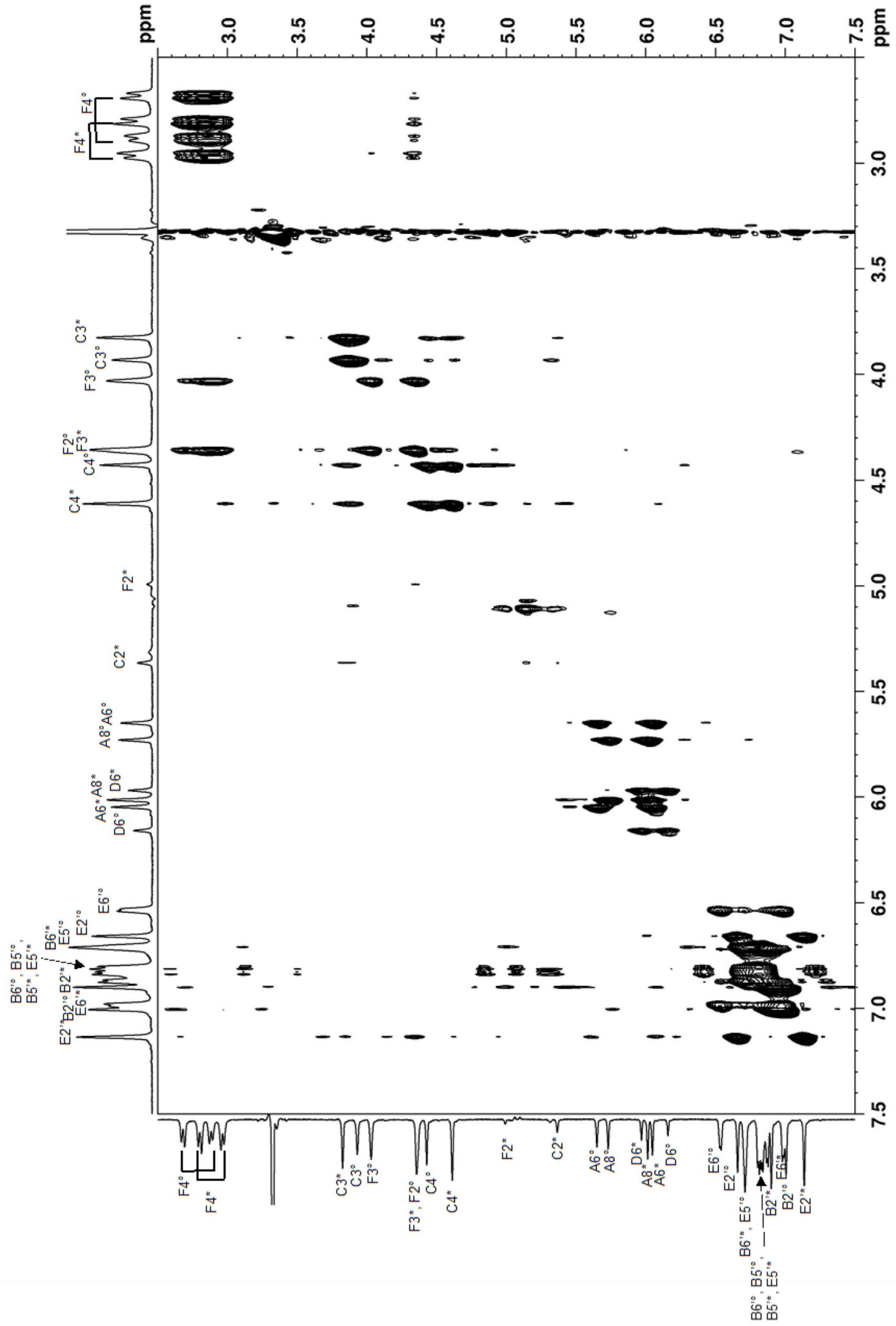

S30: ^{1}H proton and ^{13}C carbon shifts for PC B2 in methanol-d_4:D_2O (1:1) at 700 MHz and 274 K.

		H [ppm]				C [ppm]	
		major (*)		minor (°)		major (*)	minor (°)
**A**	5a					102.87	104.2
	5					154.12-157.18	154.12-157.18
	6	6.05	s	5.65	s	95.64	95.64
	7					154.12-157.18	154.12-157.18
	8	6.01	s	5.73	s	96.1	95.64
	8a					154.12-157.18	154.12-157.18
**C**	2	5.36	s	5.03	s	76.7	76.7
	3	3.82	s	3.93	s	73.1	73.1
	4	4.61	s	4.43	s	36.58	37.04
**B**	1´					132.71	132.35
	2´	6.9	m[1]	7.00	s	115.05	115.5
	3´					145.25	145.02
	4´					144.8	144.82
	5´	6.83	m[1]	6.81	m[1]	116.39	116.37
	6´	6.72	t[2]	6.84	m[1]	119.56	120
**D**	5a					100.2	100.82
	5					154.12-157.18	154.12-157.18
	6	5.97	s	6.16	s	97.45	96.1
	7					154.12-157.18	154.12-157.18
	8					107.84	109.13
	8a					154.12-157.18	154.12-157.18
**F**	2	4.99	s	4.37	s[3]	78.96	79.86
	3	4.36	s[3]	4.03	s	66.33	66.78
	4	2.96 & 2.80	dd	2.88 & 2.68	dd	29.36	29.36
**E**	1´					132.17	131.72
	2´	7.14	s	6.66	s	115.02	115.48
	3´					145.25	144.36
	4´					144.8	144.38
	5´	6.86	m[1]	6.71	t[2]	116.37	115.94
	6´	6.98	d	6.53	d	119.53	120.43

[1] overlap of B6'°, B5'*, B5'° and E5'*; [2] overlap of B6'* and E5'°; [3] overlap of F3* and F2°

S31: ^{1}H-^{1}H coupling constants for PC B2 in methanol-d_4:D_2O (1:1) at 700 MHz and 274 K.

	proton - proton coupling J [Hz]	
	major (*)	minor (°)
$J_{B5' - B6'}$	n.d.	n.d.
$J_{B6' - B5'}$	n.d.	n.d.
$J_{C2 - C3}$	n.d.	n.d.
$J_{C3 - C4}$	n.d.	n.d.
$J_{E5' - E6'}$	6.5	n.d.
$J_{E6' - E5'}$	6.8	6.4
$J_{F2 - F3}$	n.d.	n.d.
$J_{F3 - F4}$	n.d.	n.d.
$J_{F4a - F4b}$	15.4 & 16.7	15.8 & 16.6

n.d. not detectable

S32: Integrals of PC B2 and the ratio between the rotamers in methanol-d_4:D_2O (1:1) at 700 MHz and 274 K.

		Integrals [rel]				Ratio [%]	
		major (*)		minor (°)		major (*)	minor (°)
A	5a						
	5						
A	6	0.52		0.40		56	44
	7						
	8	0.57		0.44		56	44
	8a						
C	2	n.d.		n.d.		n.c.	n.c.
	3	0.83		0.71		54	46
	4	0.92		0.72		56	44
B	1´						
B	2´	1.05		1.94	i.o.[1] (2H)		
	3´						
	4´						
	5´	4.00	i.o.[2] (4H)	4.00	i.o.[2] (4H)	n.c.	n.c.
	6´	1.89	i.o.[3] (2H)	4.00	i.o.[2] (4H)	n.c.	n.c.
D	5a						
	5						
D	6	0.28		0.23		55	45
	7						
	8						
	8a						
F	2	n.d.		1.42	i.o.[4] (2H)	n.c.	n.c.
	3	1.42	i.o.[4] (2H)	0.88			
	4	1.18 &1.20		1.01 &1.04		54	46
E	1´						
E	2´	1.00		0.82		55	45
	3´						
	4´						
	5´	4.00	i.o.[2] (4H)	1.89	i.o.[3] (2H)	n.c.	n.c.
	6´	1.94	i.o.[1] (2H)	0.82		n.c.	n.c.
**mean avg.**						55 ± 1	45 ± 1

i.o. integral overlap, superscript provides number of overlapping integrals, numbers of protons are given in brackets; n.c. not calculable; n.d. not detectable due to water suppression

S33: ^{1}H – ^{1}H TOCSY spectrum (mixing time 100 ms, positive contour: black, negative contour: red) of PC B2 in 0.1M PBS/D_2O at 700 MHz and 274 K.

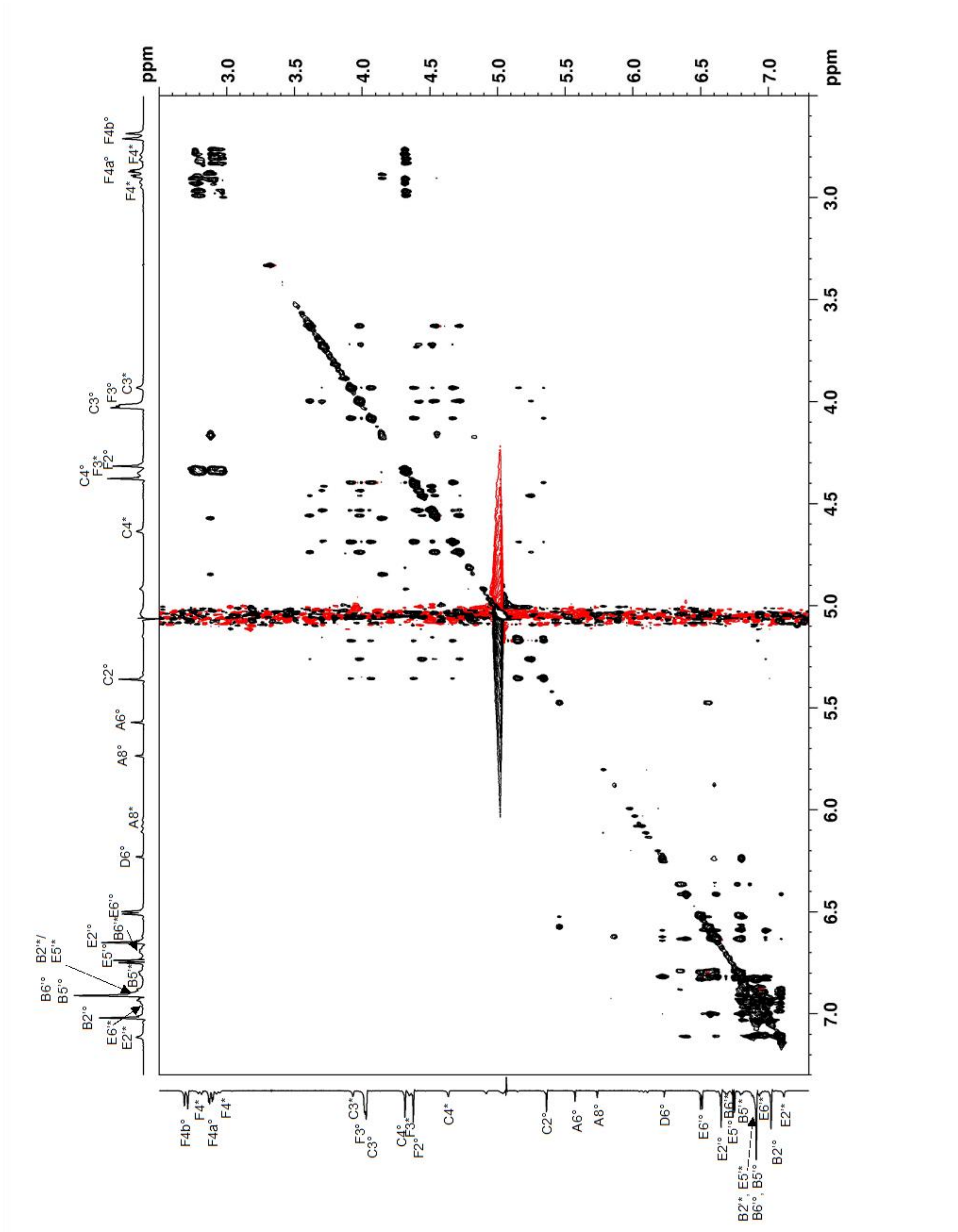

S34: Proton shifts for PC B2 in 0.1 M PBS/D_2O at 700 MHz and 274 K.

		^1H [ppm]			
		**major (*)**		**minor (°)**	
**A**	5				
	6	6.11	s	5.57	s
	7				
	8	6.08	s	5.73	s
	9				
	10				
**C**	2	n.d.		5.35	s
	3	3.93	s	4.02	s
	4	4.63	s	4.38	s
**B**	1´				
	2´	6.9	m[1]	7.02	s
	3´				
	4´				
	5´	6.8	d	6.91	s
	6´	6.69	d	6.91	s
**D**	5a				
	5				
	6	6.05	s	6.23	s
	7				
	8				
	8a				
**F**	2	n.d.		4.31	s
	3	4.35	s	4.01	d
	4	2.80 & 2.89	d	2.70 & 2.86	dd
**E**	1´				
	2´	7.11	s	6.65	s
	3´				
	4´				
	5´	6.89	m[1]	6.74	m[1]
	6´	6.94	d	6.5	m[1]

[1] overlap of B2'*, E5'*, E5'° und E6'°

S35: Proton – proton coupling constants for PC B2 in 0.1 M PBS/D_2O at 700 MHz and 274 K.

	proton - proton coupling J [Hz]	
	**major (*)**	**minor (°)**
^3J B5' - B6'	n.d.	7.6
^3J B6' - B5'	n.d.	7.6
^3J C2 - C3	n.d.	n.d.
^3J C3 - C4	n.d.	n.d.
^3J E5' - E6'	8.1	7.6
^3J E6' - E5'	8.1	6.8
^3J F2 - F3	n.d.	n.d.
^3J F3 - F4	n.d.	4.5
^3J $F4_a$ - F3	n.d.	4.9
^2J $F4_a$ - $F4_b$	16.1	17.4

n.d. not detectable

S36: Integrals of PC B2 in 0.1 M PBS/D_2O at 700 MHz and 274 K.

		integrals [rel.]				ratio [%]					
		**major (*)**		**minor (°)**		**major (*)**			**minor (°)**		
**A**	5										
	6	0.07		0.21		25			75		
	7										
	8	0.06		0.16		26			74		
	9										
	10										
**C**	2	n.d.		0.48		n.c.			n.c.		
	3	0.29		1.84	i.o.^{1} (2H)	n.c.			n.c.		
	4	0.27		0.75		26			74		
**B**	1´										
	2´	1.98	i.o.^{2} (2H)	n.d.		n.c.			n.c.		
	3´										
	4´										
	5´	0.31		1.98	i.o.^{2} (2H)	n.c.			n.c.		
	6´	0.24		1.98	i.o.^{2} (2H)	n.c.			n.c.		
**D**	5										
	6	0.04		0.13		22			78		
	7										
	8										
	9										
	10										
**F**	2	n.d.		0.67		n.c.			n.c.		
	3	0.33		1.84	i.o.^{1} (2H)	n.c.					
	4	0.36 & 0.32		1.03 & 1.00		26/24			74/76		
**E**	1´										
	2´	0.27		0.89		23			77		
	3´										
	4´										
	5´	0.35		0.81		30			70		
	6´	0.31		0.89		26			74		
**mean avg.**						25	±	2	75	±	2

i.o. integral overlap, superscript number provides number of overlapping integrals; numbers of protons are given in brackets; n.c. not calculable; n.d. not detectable

Signal assignment PC C1

S37: Proton NMR (noesygppr1d) of PC C1 in methanol-d_4:D_2O (1:1) at 700 MHz and 274 K showing shift regions of the different rings.

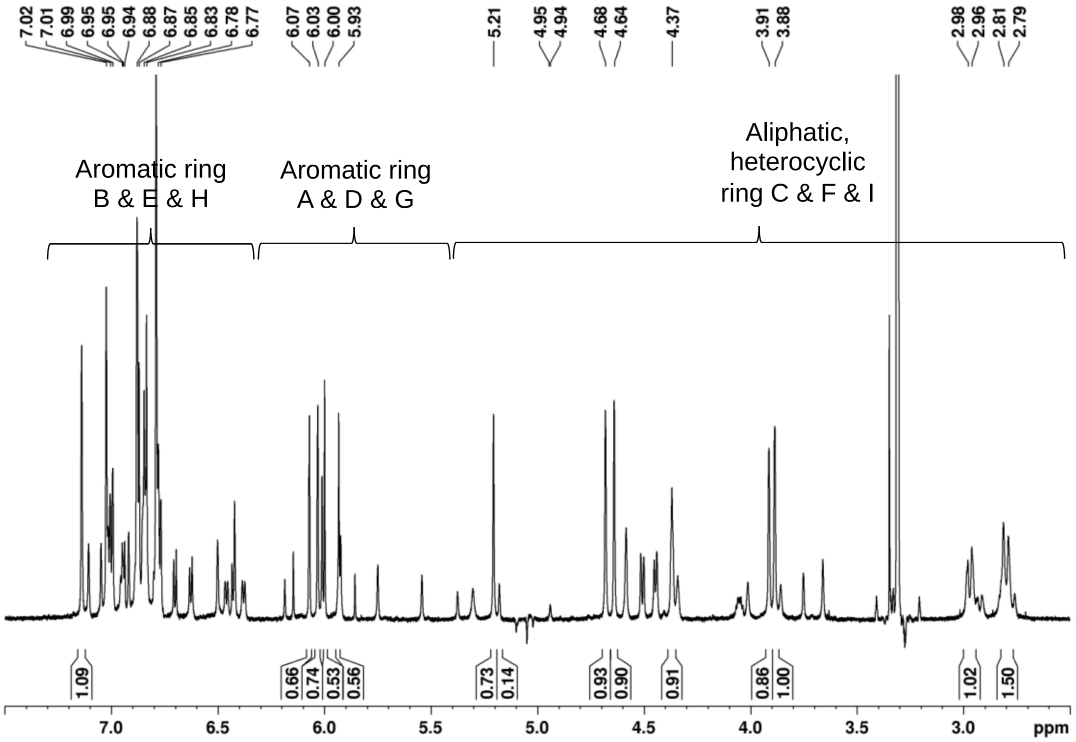

S38: Detailed signal assignment in proton spectra (noesygppr1d) of PC C1 in methanol-d_4:D2O (1:1) at 700 MHz and 274 K.

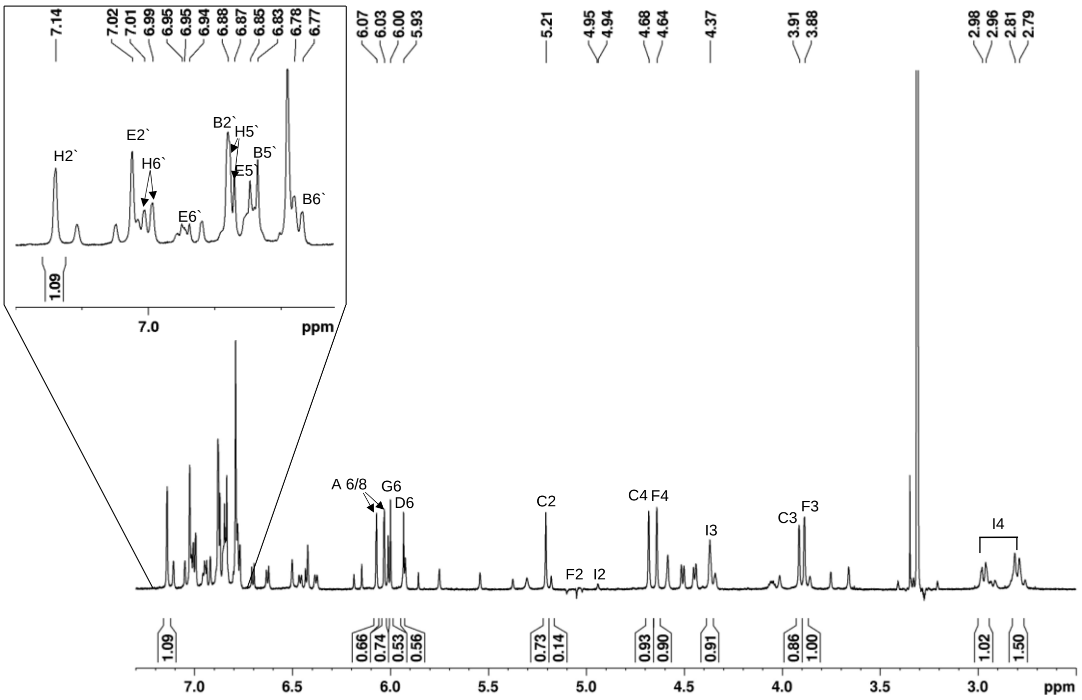

S39: Proton shifts and integrals and ^{13}C carbon shifts for PC C1 in methanol-d_4:D_2O (1:1) at 700 MHz and 274 K.

		^1H [ppm]		integral [rel.]	^{13}C [ppm]
**A**	5				154.04 - 157.33
	6	6.07 /6.03	[1]	0.66 / 0.74	95.76 & 95.96
	7				154.04 - 157.33
	8	6.07 /6.03	[1]	0.66 / 0.74	96.18 & 96.08
	9				102.82
	10				154.04 - 157.33
**C**	2	5.21	s	0.73	76.48
	3	3.91	s	0.86	73.09
	4	4.68	s	0.93	36.65
**B**	1´				132.16
	2´	6.88	m[2]	2.40 (2H)	114.86
	3´				145.01
	4´				144.83
	5´	6.83	m[3]	2.22 (2H)	116.41
	6´	6.77	d	1.15	119.31
**D**	5				154.04 - 157.33
	6	5.93	s	0.56	96.71
	7				154.04 - 157.33
	8				106.92
	9				102.41
	10				154.04 - 157.33
**F**	2	5.18		x1	76.54
	3	3.8	s	1.00	72.31
	4	4.64	s	0.90	36.61
**E**	1´				132.59
	2´	7.02	s	1.09	115.03
	3´				145.01
	4´				144.83
	5´	6.85	m[3]	2.22 (2H)	116.41
	6´	6.94	d	0.46	119.28
**G**	5				154.04 - 157.33
	6	6.00	s	0.53	97.55
	7				154.04 - 157.33
	8				108.12
	9				102.86
	10				154.04 - 157.33
**I**	2	4.94		x1	76.16
	3	4.37	s	0.91	66.33
	4	2.80 &2.97	d	1.50 & 1.02	29.27 & 29.23
**H**	1´				132.17
	2´	7.14	s	1.09	115.02
	3´				145.01
	4´				144.89
	5´	6.88	m[2]	2.40 (2H)	116.45
	6´	7.00	d	1.07	119.31

[1] can be A6 or A8; [2,3] multiplet overlay

S40: Proton – proton coupling constants for PC C1 in methanol-d_4:D_2O (1:1) at 700 MHz and 274 K.

	**proton - proton coupling J [Hz]**
$^{3}J_{B5' - B6'}$	n.d.
$^{3}J_{B6' - B5'}$	8.8
$^{3}J_{C2 - C3}$	n.d.
$^{3}J_{C3 - C4}$	n.d.
$^{3}J_{C4 - C3}$	n.d.
$^{3}J_{E5' - E6'}$	n.d.
$^{3}J_{E6' - E5'}$	n.d.
$^{3}J_{F2 - F3}$	n.d.
$^{3}J_{F3 - F4}$	n.d.
$^{3}J_{F4 - F3}$	n.d.
$^{3}J_{H5' - H6'}$	n.d.
$^{3}J_{H6' - H5'}$	8.7
$^{3}J_{I2 - I3}$	n.d.
$^{3}J_{I3 - I4}$	n.d.
$^{3}J_{I4 - I3}$	n.d.
$^{2}J_{I4_a - I4_b}$	16.7 & 13.0

n.d. not detectable

S41: $^{1}H - ^{1}H$ TOCSY spectrum (mixing time 100 ms, positive contour: black, negative contour: red) of PC C1 in 0.1M PBS/D_2O at 700 MHz and 274 K.

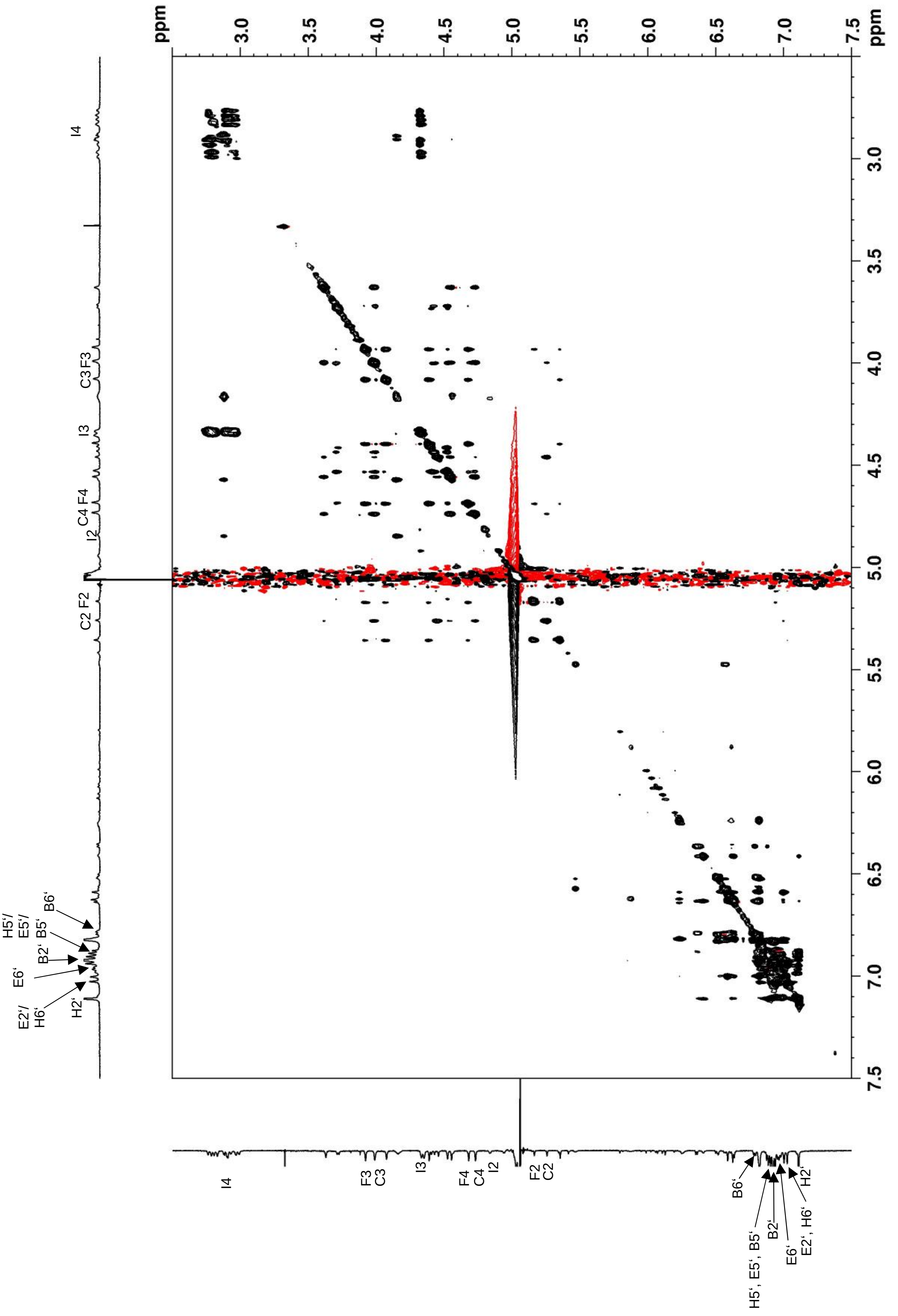

S42: Proton shifts and integrals for PC C1 in 0.1 M PBS/D_2O at 700 MHz and 274 K.

		^{1}H [ppm]		integral [rel.]	
**A**	5				
	6				
	7				
	8				
	9				
	10				
**C**	2	5.26	s	0.27	
	3	3.99	s	0.74	
	4	4.73	s	0.75	
**B**	1´				
	2´	6.91	m^{1}	3.00	i.o.^{1}
	3´				
	4´				
	5´	6.91	m^{1}	3.00	i.o.^{1}
	6´	6.79		3.00	i.o.^{2}
**D**	5				
	6				
	7				
	8				
	9				
	10				
**F**	2	5.16	s	0.29	
	3	4.07	s	1.00	
	4	4.68	s	0.73	
**E**	1´				
	2´	6.97	m^{2}	3.00	i.o.^{2}
	3´				
	4´				
	5´	6.91	m^{1}	3.00	i.o.^{1}
	6´	6.91	m^{1}	3.00	i.o.^{1}
**G**	5				
	6				
	7				
	8				
	9				
	10				
**I**	2	4.84		0.36	
	3	4.34	d	1.62	
	4	2.89 & 2.79	d, dd	1.88 & 1.27	
**H**	1´				
	2´	7.11	s	3.00	i.o.^{1}
	3´				
	4´				
	5´	6.91	m^{1}	3.00	i.o.^{1}
	6´	6.97	m^{2}	3.00	i.o.^{2}

i.o. integral overlap, superscript number provides number of overlapping integrals

Saturation transfer Difference (STD) – NMR

S43: Stacked STD spectra of 10 µM α-amylase with 1.3 mM CAT in 0.1 M PBS/ 0.04 M NaCl (D_2O/H_2O, 90/10 v/v) and an off-resonance spectrum of 1.3 mM CAT at 700 MHz and at 298 K. The saturations specified in the stacked spectra is scaled to the signals of the aromatic ring B.

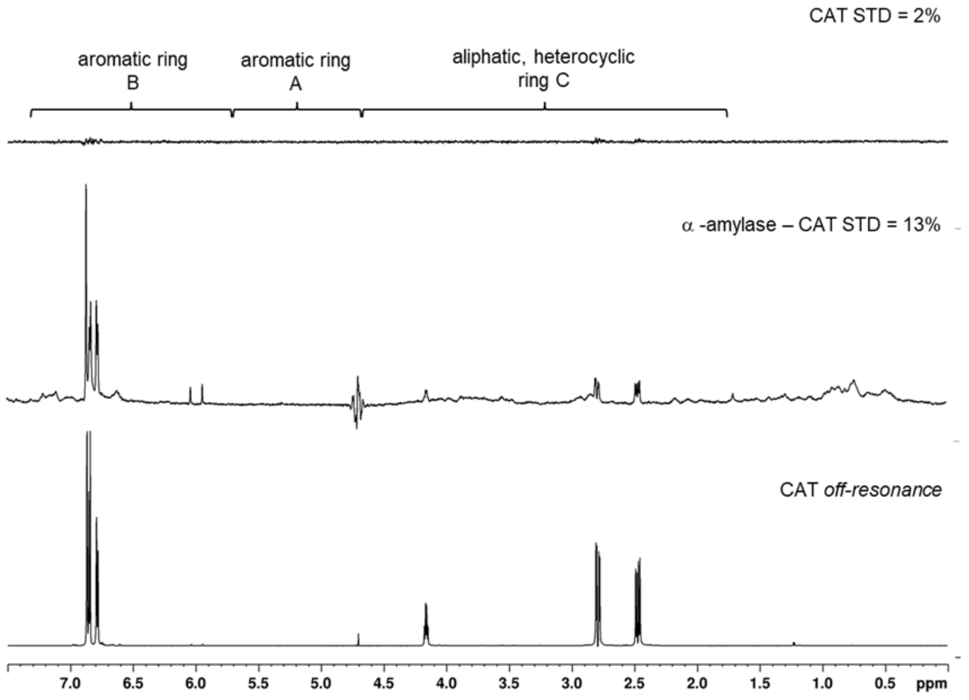

S44: Stacked STD spectra of 10 µM α-amylase with 0.9 mM PC B1 in 0.1 M PBS/ 0.04 M NaCl (D_2O/H_2O, 90/10 v/v) and an off-resonance spectrum of 0.9 mM PC B1 at 700 MHz and at 298 K. The saturations specified in the stacked spectra is scaled to the signals of the aromatic rings B and E.

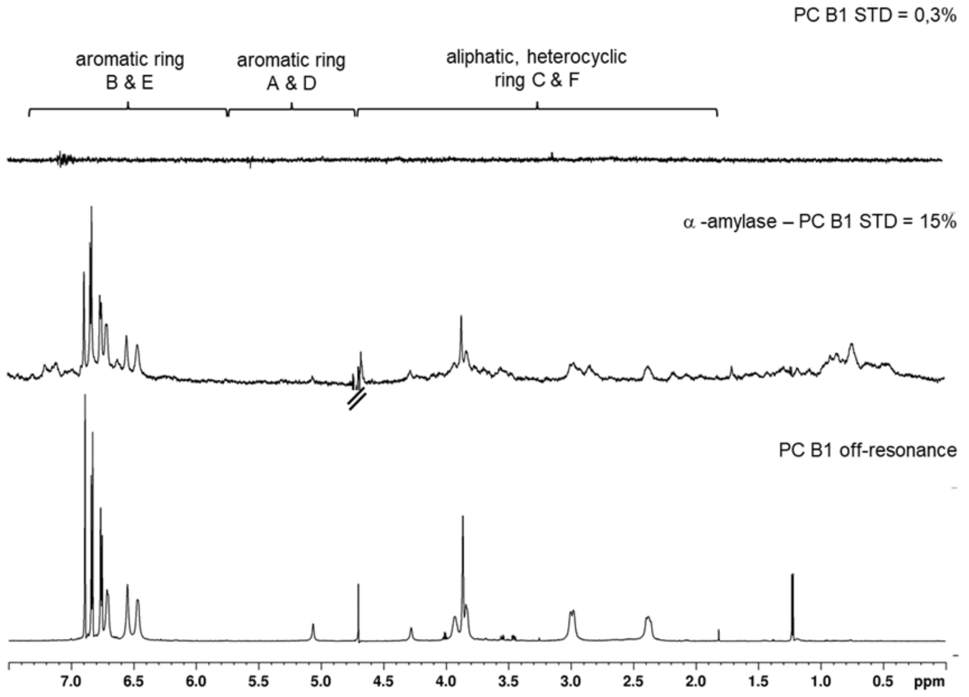

S45: Stacked STD spectra of 10 µM α-amylase with 0.9 mM PC B2 in 0.1 M PBS/ 0.04 M NaCl (D_2O/H_2O, 90/10 v/v) and an off-resonance spectrum of 0.9 mM PC B2 at 700 MHz and at 298 K. The saturations specified in the stacked spectra is scaled to the signals of the aromatic rings B and E.

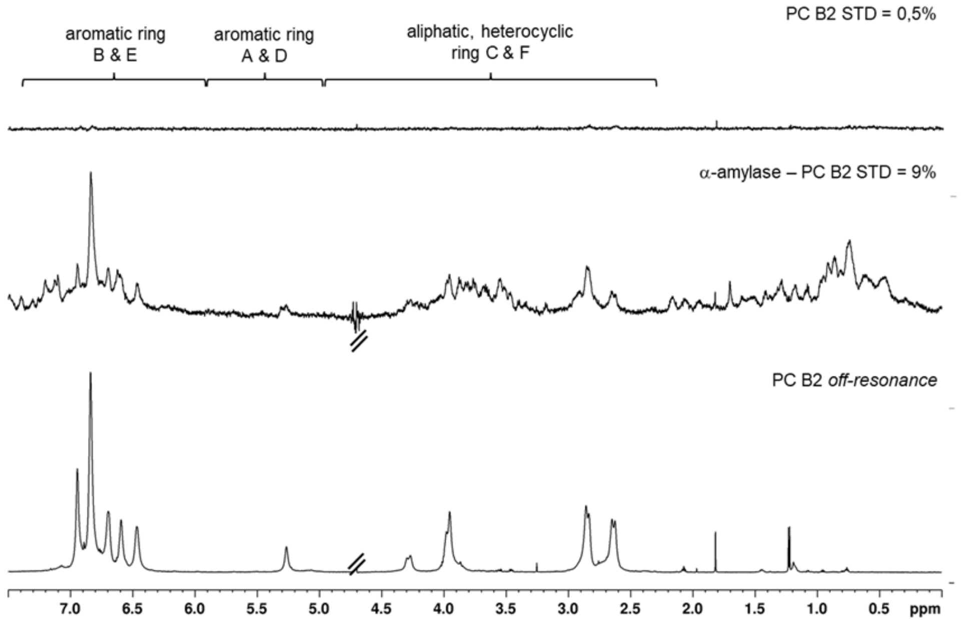

S46: Stacked STD spectra of 10 µM α-amylase with 1.0 mM PC C1 in 0.1 M PBS/ 0.04 M NaCl (D_2O/H_2O, 90/10 v/v) and an off-resonance spectrum of 1.0 mM PC C1 at 700 MHz and at 298 K. The saturations specified in the stacked spectra is scaled to the signals of the aromatic rings B, E and H.

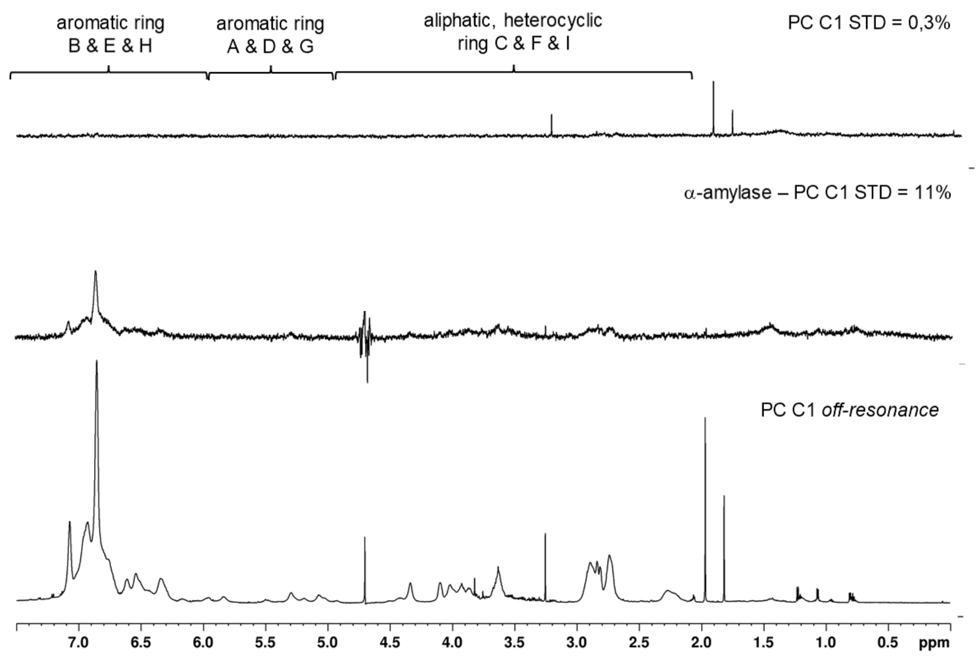

Proton NMR of PC C1 in the presence of α-amylase

S47: Full proton spectra (zgesgp) of PC C1 and changes in intensities by addition α-amylase at 275 K in 0.1 M phosphate buffer (D_2O:H_2O 9:1, 0.04 M NaCl, pH 7).

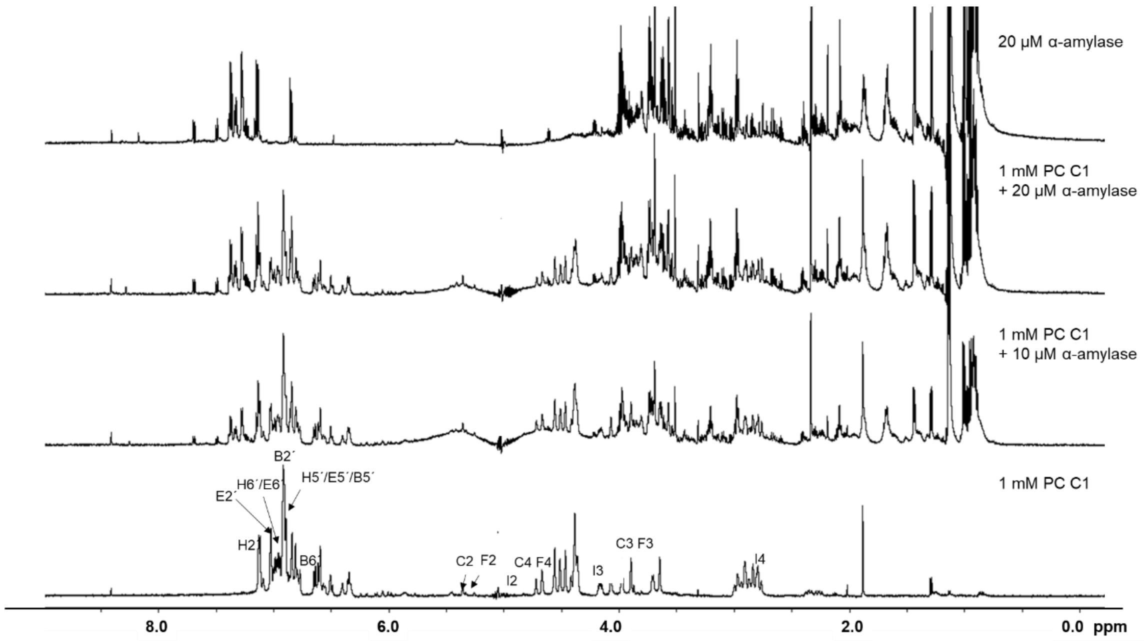

S48: Overlay of PC C1 and α-amylase proton spectra (zgesgp) and mixtures thereof at 275 K in 0.1 M phosphate buffer (D_2O:H_2O 9:1, 0.04 M NaCl, pH 7). The spectral range to calculate the proportion of PC C1 aggregated with α-amylase is indicated.

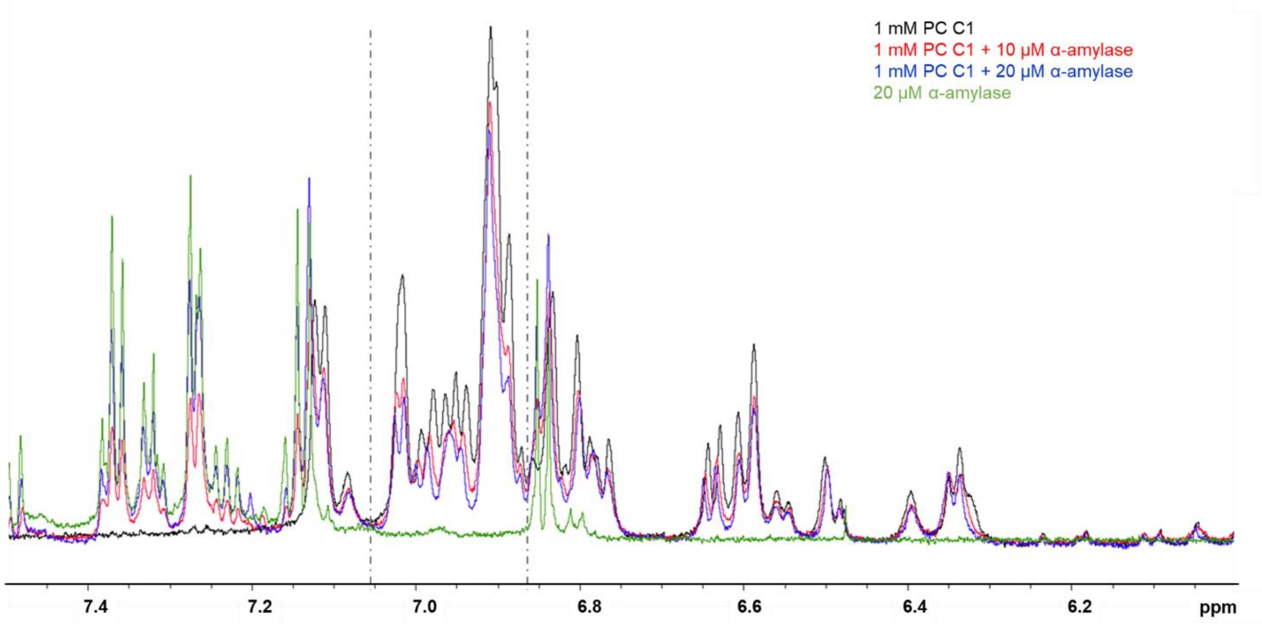

Determination of change in secondary structure of α-amylase by CD spectroscopy

S49: Calculated secondary protein structures of the different samples (90 µM α-amylase, 600 µM polyphenol).

solution	α-helix [%]	β-sheet [%]	β-turn [%]	random coil [%]
α-amylase	8	32	12	48
α-amylase + EC	14	23	15	48
α-amylase + PC B2	14	23	15	48

Inhibition studies of flavan-3-ols with α-Amylase

S50: Thermogram of recurrent single injection experiment with starch (1.1 g/L (5 mM) per injection) titrated into α-amylase (12 nM) at 310 K (left) and overlay of injections (right) (control).

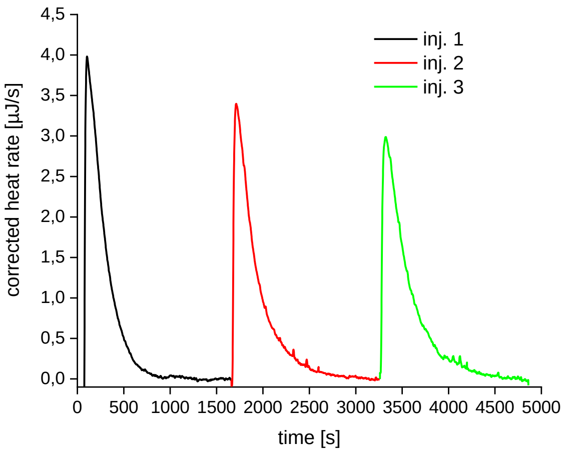

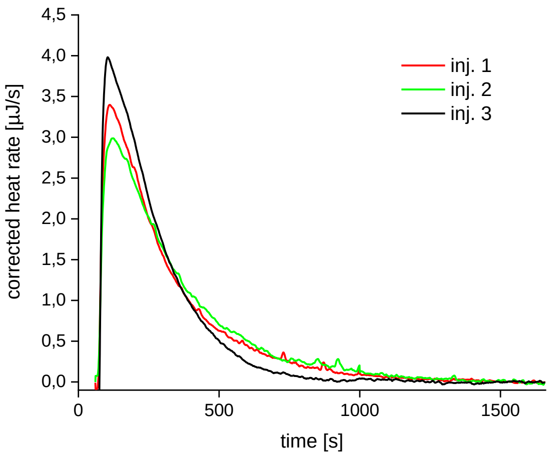

S51: Changes in inhibition parameters calculated for the flavan-3-ols (100 µM) with increasing impact of product inhibition from injection (inj.) 1 to 3.

	v_{max} [%]			K_m [mM]		
	inj. 1	inj. 2	inj. 3	inj. 1	inj. 2	inj. 3
inhibitor	mean	mean	mean	mean	mean	mean
control	100	100	100	2.33±0.10	2.79±0.09	2.87±0.35
EC	78±4	77±5	76±3	2.55±0.09	2.80±0.20	2.79±0.04
CAT	79±6	78±5	79±2	2.50±0.37	2.72±0.11	2.61±0.05
PC B1	66±9	65±8	70±1	2.65±0.25	2.55±0.15	2.54±0.18
PC B2	62±4	62±6	61±7	2.31±0.08	2.67±0.19	2.84±0.14
PC C1	50±4	50±5	59±2	2.44±0.18	2.48±0.09	2.38±0.06

	K_{ic} [µM]			K_{iu} [µM]		
	inj. 1	inj. 2	inj. 3	inj. 1	inj. 2	inj. 3
inhibitor	mean	mean	mean	mean	mean	mean
control		12302±1653	15434±3530		24991±2585	26715±9821
EC	253±47	305±162	291±80	385±82	389±41	330±52
CAT	356±209	447±151	458±152	431±217	403±134	399±54
PC B1	155±79	216±65	539±86	219±91	208±78	240±14
PC B2	177±39	188±34	202±73	173±26	195±57	165±49
PC C1	94±26	143±56	395±78	103±16	102±19	145±14

S52: Thermogram of starch conversion (1.1 g/L (5mM), 310 K) after incubation of 12 nM α-amylase with 0 µM (control, black), 100 µM (red) and 105 µM (blue) PC C1.

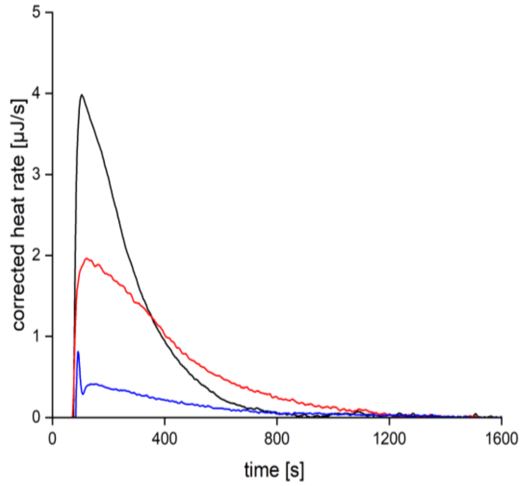

S53: Correlation of IC50 values with the molecular weight

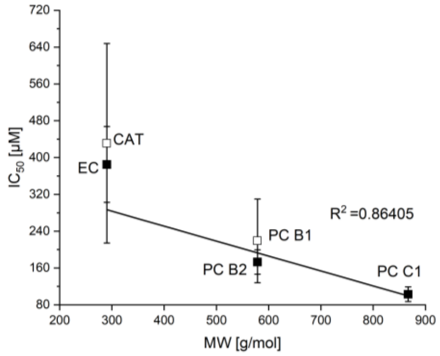

Supplement: Supplementary file 1 — jf4c13178_si_001.pdf [file jf4c13178_si_001.pdf]
